# Contributions to the Dynamic Regime Behavior of a Bionic Leg Prosthesis

**DOI:** 10.3390/biomimetics8050414

**Published:** 2023-09-06

**Authors:** Marius-Valentin Drăgoi, Anton Hadăr, Nicolae Goga, Florin Baciu, Amado Ștefan, Lucian Ștefăniță Grigore, Damian Gorgoteanu, Cristian Molder, Ionica Oncioiu

**Affiliations:** 1 Department of Strength of Materials, Faculty of Industrial Engineering and Robotics, Politehnica University of Bucharest, 060042 București, Romania; marius.dragoi@upb.ro (M.-V.D.); anton.hadar@upb.ro (A.H.); florin.baciu@upb.ro (F.B.); 2 Academy of Romanian Scientists, 3 Ilfov Street, Sector 5, 050045 Bucharest, Romania; 3 Technical Sciences Academy of Romania, 26 Dacia Boulevard, Sector 1, 030167 Bucharest, Romania; 4 Faculty of Engineering in Foreign Languages, Politehnica University of Bucharest, 060042 București, Romania; nicu.goga@upb.ro; 5 Department of Integrated Aviation Systems and Mechanics, Faculty of Aircraft and Military Vehicles, “Ferdinand I” Military Technical Academy, 050141 Bucharest, Romania; amado.stefan@mta.ro; 6 Center of Excellence in Robotics and Autonomous Systems—CERAS, “Ferdinand I” Military Technical Academy, 050141 Bucharest, Romania; lucian.grigore@mta.ro (L.Ș.G.); damian.gorgoteanu@mta.ro (D.G.); cristian.molder@mta.ro (C.M.); 7 Department of Informatics, Faculty of Informatics, Titu Maiorescu University, 040051 Bucharest, Romania

**Keywords:** design, biped walking, dynamic, stresses, digital image correlation, mechanical properties of PLA, finite element analysis

## Abstract

The purpose of prosthetic devices is to reproduce the angular-torque profile of a healthy human during locomotion. A lightweight and energy-efficient joint is capable of decreasing the peak actuator power and/or power consumption per gait cycle, while adequately meeting profile-matching constraints. The aim of this study was to highlight the dynamic characteristics of a bionic leg with electric actuators with rotational movement. Three-dimensional (3D)-printing technology was used to create the leg, and servomotors were used for the joints. A stepper motor was used for horizontal movement. For better numerical simulation of the printed model, three mechanical tests were carried out (tension, compression, and bending), based on which the main mechanical characteristics necessary for the numerical simulation were obtained. For the experimental model made, the dynamic stresses could be determined, which highlights the fact that, under the conditions given for the experimental model, the prosthesis resists.

## 1. Introduction

Over time, prostheses made to replace missing limbs, or to replace missing parts of a person’s limbs, have kept pace with the evolution of technology. Currently, to manufacture a prosthesis, the design is carried out down to the smallest detail of the parts used in the assembly of the final product. According to the World Health Organization, approximately 0.5% of the global population uses or requires a prosthesis or orthosis. Of the 40 million patients requiring specialist treatment globally, approximately two-to-four times as many people attend services dedicated to orthotic treatment [1,2].

The goal of modern prostheses is to replicate [3] the function of the replaced limb or organ in the most capable and discreet way possible. However, even the most advanced transtibial prostheses available today only passively adjust the ankle position during the swing phase of the gait, and return some of the user’s gravitational input [4,5]. To greatly improve the quality of life of a transtibial amputee, new technologies and approaches must be used to create a robotic ankle prosthesis that can perform similarly to, if not better than, the equivalent of the able-bodied human ankle. An example of a bionic prosthetic lower limb was developed by the US Army through the Medical Research and Materiel Command (USAMRMC) [6]. The project developed by USAMRMC is called SPARKy (Spring Ankle with Regenerative Kinetics), and aims to bring full working ankle function to transtibial amputees, especially those injured serving in the military who want to be able to return to active duty.

Another active transfemoral prosthesis that allows the reproduction of average walking at ground level is the Cyberlegs Beta-Prosthesis [7]. This prosthesis consists of an elastic actuator in series with a parallel spring, to store energy captured during walking at the start of a step, and release it during push-off, to provide extra energy [8]. Although it provides power while walking, this system is heavy and bulky. In general, transfemoral prostheses are passive and modular, and can generate articulated movement. In support of this idea are the prosthetic systems developed by Staros SACH [9], Hafner ESR [10], Mauch [11], and OttoBock emPOWER [12]. It has been shown that these passive propulsion systems are not reliable long-term solutions [13]. We also note the following prostheses: the Vanderbilt prosthesis that has a spring in the ankle, and works during plantar flexion in parallel with the motors [14]; the CSEA knee, which is based on a friction clutch whose purpose is to lock the various elastic elements, to develop angular torque depending on how the heel is placed on the ground [15]; and the ETH/Delft knee, which combines actuator stiffness with real joint stiffness [16].

These systems provide energy-efficient walking kinematics. The role of the springs is to reduce energy consumption by reducing the holding torque. As a working principle, the engagement of the spring depends only on the angle of the ankle. For this reason, active ankle and knee actuation systems offer additional benefits. These benefits include a reduction in the load on the unaffected leg, through the use of an electric ankle [17].

Active prosthetic systems [18,19] are kinematically and dynamically superior to passive systems [20,21,22]. However, they are taxing in terms of the electrical energy storage capacity [23,24], which affects the operating autonomy [25].

We appreciate that there is a similarity regarding the concept described in [26], but in our model, we make improvements related to the kinematic and dynamic models. From an analytical–numerical point of view, the bionic prosthesis solution includes the operating laws of the prosthesis, which respect the anatomy of a human leg.

The bionic prosthesis is made via a rigid body mechanism, equipped with electric actuators to perform joint rotations [27,28,29]. The main challenges will be in the design and realization of the joints and the skeleton.

**Figure 1:**
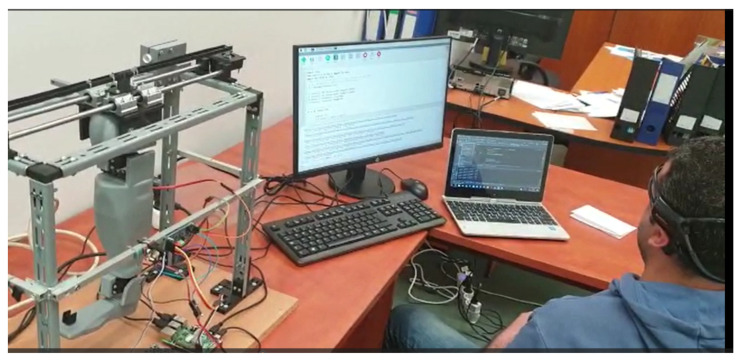
A prosthesis operated by means of a neural headset: neurally actuated prosthetic prosthesis (NAPP).

The purpose of this work is to highlight the dynamic characteristics of a lower limb prosthesis, as an alternative to existing passive or mixed systems. The proposed system has been realized at the level of an experimental laboratory model, as can be seen in Figure 1.

The prosthesis consists of a knee actuated via a servomotor, and an ankle also actuated via a servomotor. The solution, to be developed in the future, is intended to be used as a prosthesis for people whose lower limb (from the knee down) has been amputated, or to equip a medium-sized humanoid robot. To set the prosthesis in motion, a system/frame was made that also had a stepper motor for longitudinal movement. An actuation was also performed on the two servomotors, with the help of a neural headset. The primary intention was to create a prosthetic prosthesis that could be operated with the help of brain waves.

The main contributions of the paper consist of:

- The creation of a prosthesis of the lower part of the leg, an experimental model operated with electric motors (brushless);
- creating a command and control system that allows the foot to be operated with the help of a neural headset;
- creating the framework concept for the design of a foot prosthesis so that, in the later stage of development, we can create a prosthesis that respects the real configuration of the foot, and is capable of responding to real mechanical demands;
- creating the analytical model that describes the kinematic and dynamic operation of such a prosthesis;
- the creation of the numerical model, which allows us to highlight the dynamic demands of the prosthesis;
- obtaining the areas where tension concentrators appear, something that will be solved via a new configuration/structure for the future prosthesis;
- highlighting the limitations of the experimental model created.

The training framework for the prosthetic system (bionic leg) was made in the Laboratory of the Materials Resistance Department of the POLITEHNICA University in Bucharest, in collaboration with the Center of Excellence in Robotics and Autonomous Systems (CERAS). The developed solution is an experimental model, validated in the research laboratory. Although the analyzed system is a consumer of electricity, in the future, a compartment for Li-ion batteries will be made in the calf, with the possibility of having a portable battery on the operator.

The paper is structured as follows: Section 2 discusses how the instrumentation was used to test the operation of the bionic foot assembly, in order to determine the correlation between longitudinal axis displacement and vibrations in the foot and ankle area. Also presents the results obtained analytically regarding the kinematics and dynamics of the foot. Section 3 presents the results: experimental measurements, mechanical test results of the test pieces and the numerical analysis, and describes the comparative analysis of the results obtained experimentally, and those obtained from the numerical analysis. Section 4 presents the conclusions, and the potential options for further development.

## 2. Materials and Methods

**Figure 2:**
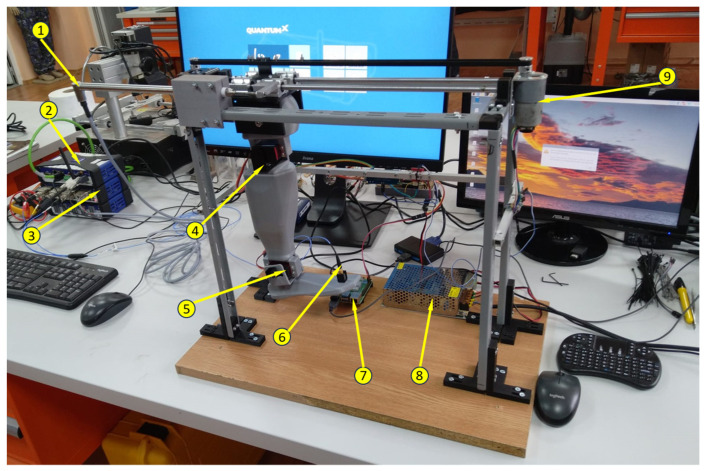
The bionic leg experimental setup: 1. HBM’s WA200-series displacement sensor #260510079; 2. QuantumX CX22B-W computer S/N F0F9578297C8; 3. strain gauge MX840B S/N 0009E520A43; 4. Servomotor K-Power HBL090 ball joint; 5. K-Power HBL090 ankle actuator; 6. Accelerometer PCB Piezotronics 356A43 S/N LW348378; 7. Raspberry Pi4 model B 4GB RAM; and 8. power supply; 9. Motor JGB37-520 (12 V, 1:90, 107 RPM).

The experimental tests carried out during this research consisted of the determination of the material characteristics, the displacement along the longitudinal axis, and the vibrations at two points on the foot. The experimental setup for testing the bionic leg is shown in Figure 2.

### 2.1. Materials Used

The component elements of the leg were fabricated using the method of 3D printing from PLA (polylactic acid or polylactide) [30]. PLA is a bioabsorbable biopolymer produced from non-toxic renewable raw material [31]. The solution chosen regarding the material from which the leg is made, at this point in the research, also took into account the fact that PLA is considered good for medical applications [32]. We make this statement because, at a later stage of development, the bionic leg will come into contact with the human body. The physical properties of PLA, according to [33], are shown in Table 1.

The PLA used is soluble in chloroform, methylene chloride, etc., and degrades via hydrolysis after exposure to a moist environment.

**Figure 3:**
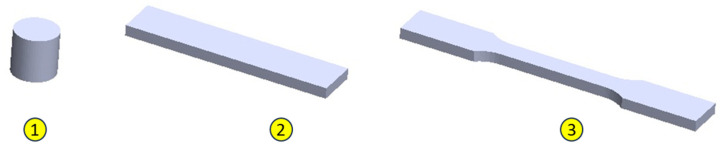
Specimens for determining the physical/mechanical characteristics of the PLA used in the construction of the bionic leg: 1. compression test specimen; 2. tensile test specimen; 3. test specimen for bending stress.

To validate the physical–mechanical properties, three types of samples were made, to test the material under compression, stretching, and bending stresses (Figure 3). The material characteristics can be found in Table 2.

After 10 samples were printed for each type of test, they were measured, and the dimensions in Table 3, Table 4 and Table 5 were obtained.

### 2.2. The Technology for Making the Leg

**Figure 4:**
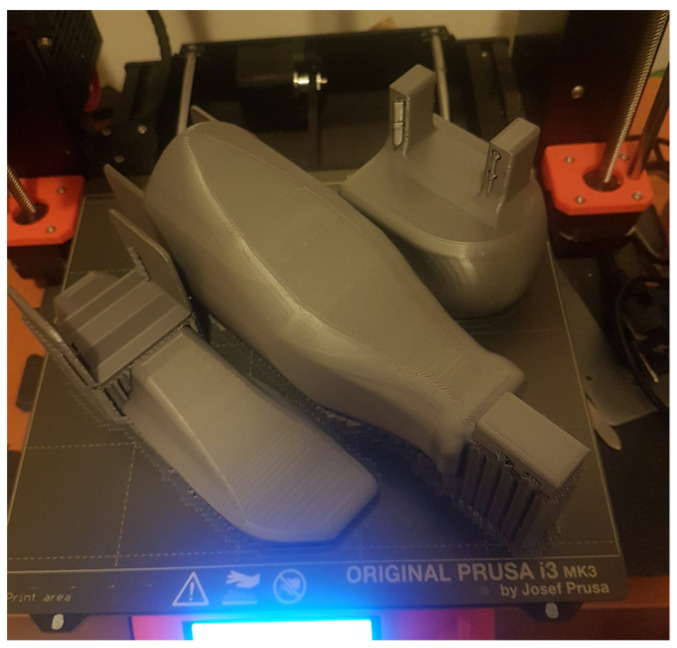
The 3D printing of the three component elements of the bionic leg.

**Figure 5:**
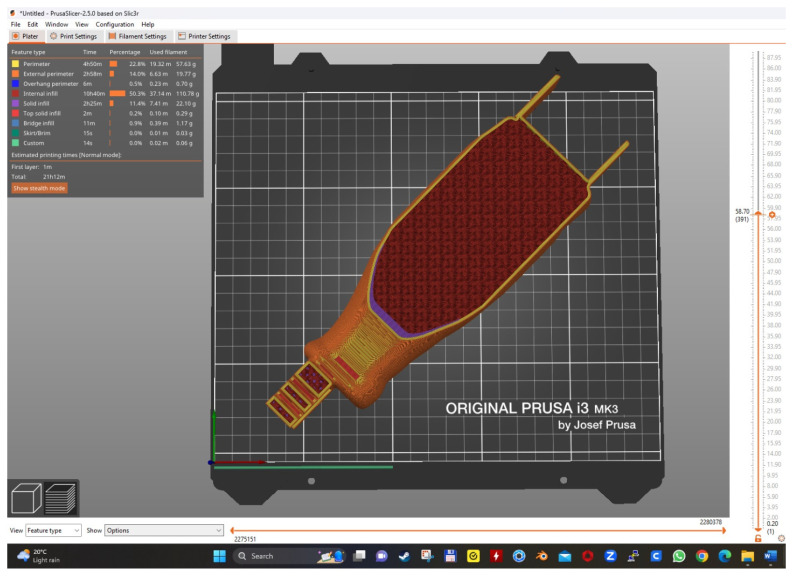
An example of the 3D printing of the calf, with characteristics according to Table 2.

The component elements of the bionic prosthesis after the design phase were processed in a specific language for the PRUSA i3 MK3 3D printer (Figure 4 and Figure 5).

**Figure 6:**
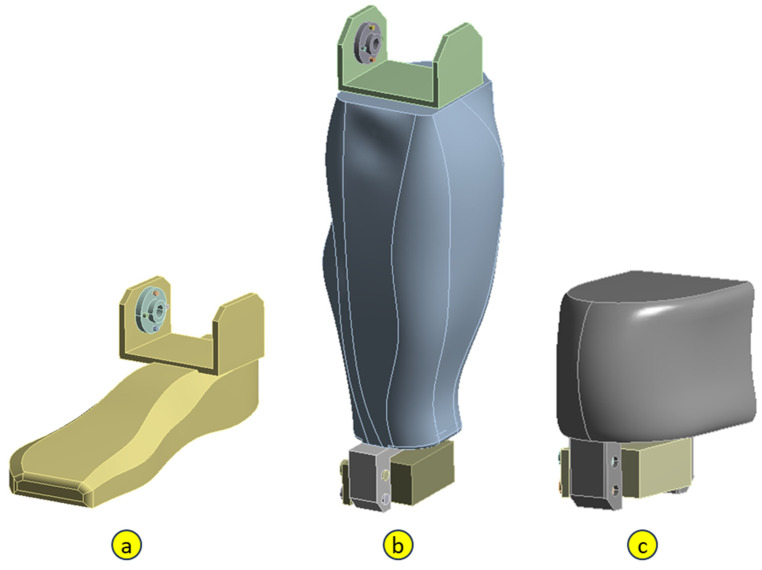
The component elements of the bionic foot: a. sole; b. calf; c. knee.

As already stated, the purpose of the research is to determine the resistance capacity of a prototype foot made using additive technology (3D printing from PLA) to withstand the dynamic demands produced by its movement, according to an imposed law. The component parts of the foot are the sole (Figure 6a), the lower leg (Figure 6b), and the knee (Figure 6c).

From a constructive/assembly point of view, we have the following:

1. Sole:
  - The foot (monobloc piece made from PLA);
  - The motor attachment bushing, positioned next to the ankle (aluminum);
  - Fixing screws between the bushing and the foot (steel);
2. Calf:
  - The gamba (monobloc part made of PLA);
  - The sole drive actuator (considered aluminum);
  - Actuator mounting bolts (steel);
  - The motor mounting bush, positioned next to the knee (aluminum);
  - Clamping screws between the bushing and the shank (steel);
3. Knee:
  - The knee (monobloc piece from PLA);
  - The calf actuator (considered aluminum);
  - Actuator mounting bolts (steel).

**Figure 7:**
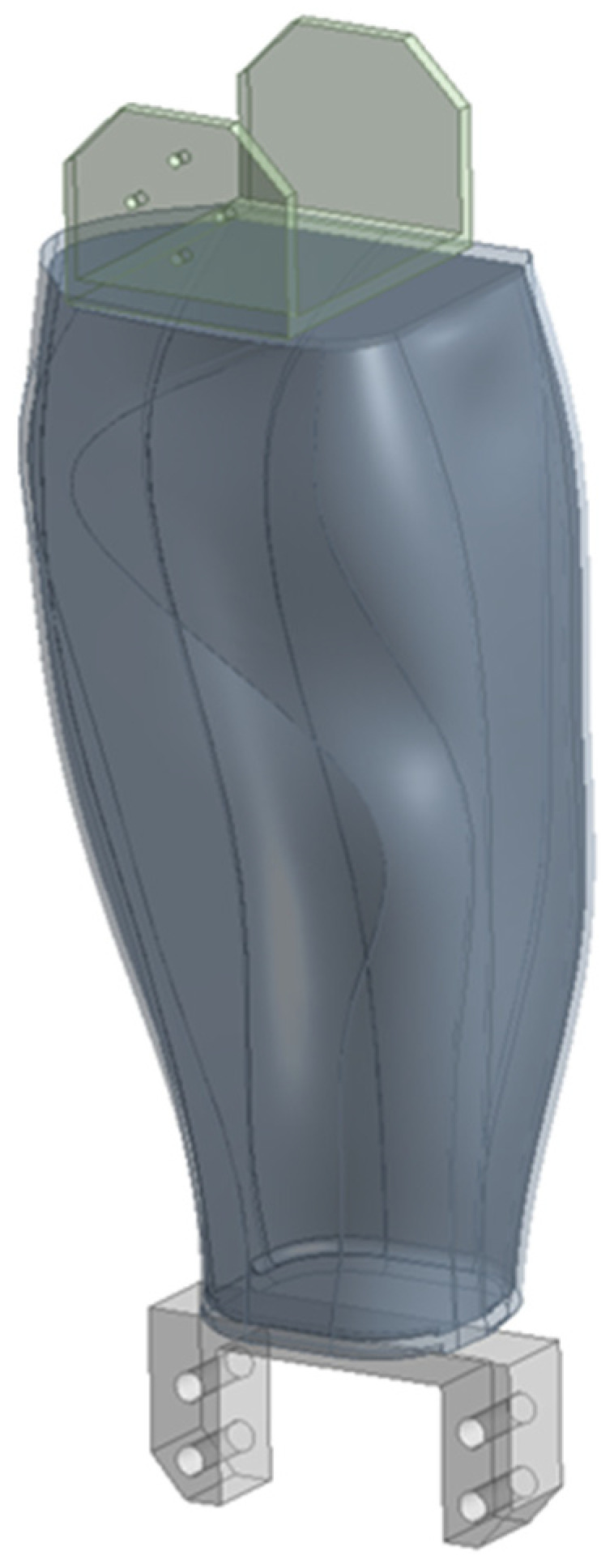
The calf geometry, with a separately molded interior.

The inside of the calf and foot were printed with 30% infill, and the outer walls were printed with 1 mm thickness. Due to this aspect, the two components were geometrically modeled separately. Figure 7 shows the outside of the calf (transparent) printed with 100% fill, and the inside (opaque) printed with 30% fill.

The movement of the experimental model is to be controlled with the help of an interface (neural helmet) that reads and interprets brain impulses. As the interface is in the design and testing phase, in this phase, there is no problem with stepping on an obstacle, so the forces that apply to the experimental model are weight and inertial forces.

### 2.3. Methods

#### 2.3.1. Kinematics

**Figure 8:**
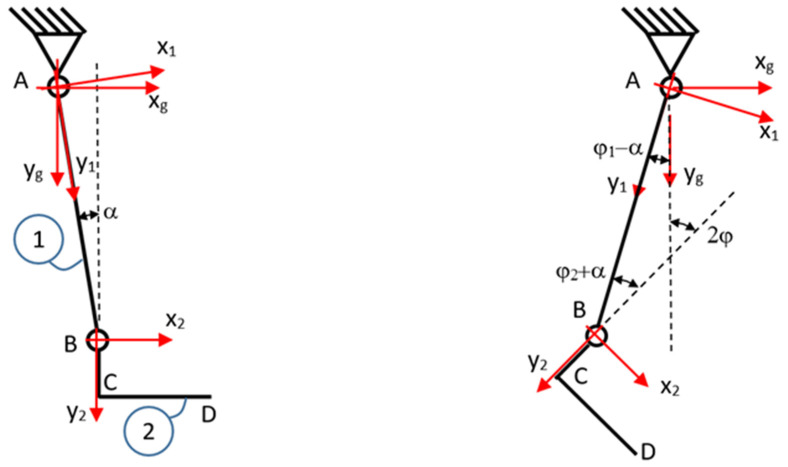
Schematic of the foot in the initial position, and at one point in time, t.

**Figure 9:**
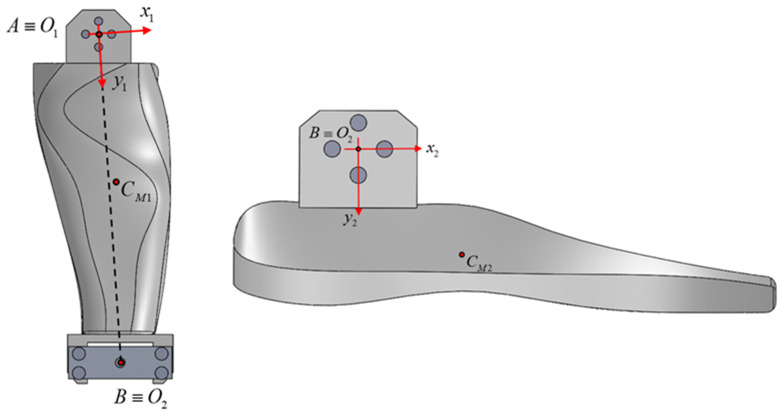
The position of the local coordinate systems.

To estimate the forces in the leg joints, linear motion laws for the rotation angle of the output shaft were imposed on the actuators. Thus, in an interval of 5 s, the shaft of each servomotor rotates by an angle of 20 degrees. The resulting kinematics are simple if a schematization is adopted, as in Figure 8 and Figure 9. According to this schematization, the velocities and accelerations of the prosthesis components are determined.

The calf is marked with 1, the sole with 2, the joint between the knee and calf with A, and the joint between the calf and sole with B. The global system is denoted $\left(x_{g},y_{g}\right)$, and the local systems related to the calf and sole are denoted $\left(x_{1},y_{1}\right)$ and $\left(x_{2},y_{2}\right)$ (Table 6). Between the line of the calf joints, for the considered vertical position, there is an angle of α=2.628°.

The position vector of the joint in B is given by the following vector relation:

(1) $${\vec{r}}_{B}=-L_{1}\cdot \sin\left(\varphi_{1}-\alpha \right)\cdot {\vec{i}}_{g}+L_{1}\cdot \cos\left(\varphi_{1}-\alpha \right)\cdot {\vec{j}}_{g},$$

where $\left(\varphi_{1}-\alpha \right)$ is the angle between the vertical and segment AB, measured clockwise; $L_{1}=204\;\mathrm{mm}$ is the length of segment AB.

The law of variation of angle $\varphi_{1}$ is $\varphi_{1}\left(t\right)=t$, being the same as $\varphi_{2}$, and ${\vec{i}}_{g}$ and ${\vec{j}}_{g}$ are the vertices of the global coordinate system.

Via derivation with respect to time, the velocity results and the second-order derivative give the expression of the acceleration. As the derivative of the angle is the angular velocity, it is noted that it is constant over time: $\omega_{1}={\dot{\varphi }}_{1}=20\cdot \frac{\pi }{180\cdot 5}$. For simplification, we write $\varphi =\varphi_{1}=\varphi_{2}$; consequently, $\omega_{1}={\dot{\varphi }}_{1}=\omega_{2}={\dot{\varphi }}_{2}=\omega$.

The vector expressions for velocity and acceleration are as follows:

(2) $${\vec{v}}_{B}=-L_{1}\cdot \omega_{1}\cdot \cos\left(\varphi_{1}-\alpha \right)\cdot {\vec{i}}_{g}-L_{1}\cdot \omega_{1}\cdot \sin\left(\varphi_{1}-\alpha \right)\cdot {\vec{j}}_{g},$$

(3) $${\vec{a}}_{B}=L_{1}\cdot \omega_{1}^{2}\cdot \sin\left(\varphi_{1}-\alpha \right)\cdot {\vec{i}}_{g}+L_{1}\cdot \omega_{1}^{2}\cdot \cos\left(\varphi_{1}-\alpha \right)\cdot {\vec{j}}_{g},$$

For the center of mass of the calf, the position relative to the local system is given by

(4) $${\vec{r}}_{CM1}=x_{CM1}{\vec{i}}_{1}+y_{CM1}{\vec{j}}_{1},$$

Using the relationship between the mobile and the fixed system’s calf-related feeders,

(5) $${\vec{i}}_{1}={\vec{i}}_{g}\cdot \cos\left(\varphi -\alpha \right)+{\vec{j}}_{g}\cdot \sin\left(\varphi -\alpha \right),$$

(6) $${\vec{j}}_{1}=-{\vec{i}}_{g}\cdot \sin\left(\varphi -\alpha \right)+{\vec{j}}_{g}\cdot \cos\left(\varphi -\alpha \right),$$

which results in the position vector with respect to the fixed system:

(7) $$\begin{array}{l} {\vec{r}}_{CM1}=\left(x_{CM1}\cos\left(\varphi -\alpha \right)-y_{CM1}\sin\left(\varphi -\alpha \right)\right){\vec{i}}_{g}+\\ +\left(x_{CM1}\sin\left(\varphi -\alpha \right)+y_{CM1}\cos\left(\varphi -\alpha \right)\right){\vec{j}}_{g} \end{array},$$

The acceleration of the center of mass is obtained via differentiating twice with respect to time:

(8) $$\begin{array}{l} {\vec{a}}_{CM1}=\omega^{2}\left(-x_{CM1}\cos\left(\varphi -\alpha \right)+y_{CM1}\sin\left(\varphi -\alpha \right)\right){\vec{i}}_{g}+\\ +\omega^{2}\left(-x_{CM1}\sin\left(\varphi -\alpha \right)-y_{CM1}\cos\left(\varphi -\alpha \right)\right){\vec{j}}_{g} \end{array},$$

For the center of mass of the sole, we determine the position vector from the following relationship:

(9) $$\begin{array}{l} {\vec{r}}_{CM2}=\vec{AB}+\vec{BC_{M2}}=\\ =-L_{1}\cdot \sin\left(\varphi_{1}-\alpha \right)\cdot {\vec{i}}_{g}+L_{1}\cdot \cos\left(\varphi_{1}-\alpha \right)\cdot {\vec{j}}_{g}+x_{CM2}{\vec{i}}_{2}+y_{CM2}{\vec{j}}_{2} \end{array},$$

The coordinate system related to the sole, with the pourers ${\vec{i}}_{2}$ and ${\vec{j}}_{2}$, is rotated relative to the fixed coordinate system by the angle $\varphi_{1}+\varphi_{2}=2\varphi$. The connection between the mobile and the fixed systems is given by the following relations:

(10) $${\vec{i}}_{2}={\vec{i}}_{g}\cdot \cos\left(2\varphi \right)+{\vec{j}}_{g}\cdot \sin\left(2\varphi \right),$$

(11) $${\vec{j}}_{2}=-{\vec{i}}_{g}\cdot \sin\left(2\varphi \right)+{\vec{j}}_{g}\cdot \cos\left(2\varphi \right),$$

Substituting vertices into the position vector relation of the center of mass $C_{M2}$, we obtain:

(12) $$\begin{array}{l} {\vec{r}}_{CM2}=\left(-L_{1}\cdot \sin\left(\varphi -\alpha \right)+x_{CM2}\cos\left(2\varphi \right)-y_{CM2}\sin\left(2\varphi \right)\right)\cdot {\vec{i}}_{g}+\\ +\left(L_{1}\cdot \cos\left(\varphi -\alpha \right)+x_{CM2}\sin\left(2\varphi \right)+y_{CM2}\cos\left(2\varphi \right)\right)\cdot {\vec{j}}_{g} \end{array},$$

By deriving the speed results with respect to time, and by deriving twice, the acceleration is obtained. The vector expressions with respect to the global system are:

(13) $$\begin{array}{l} {\vec{v}}_{CM2}=\omega \left(-L_{1}\cdot \cos\left(\varphi -\alpha \right)-2x_{CM2}\sin\left(2\varphi \right)-2y_{CM2}\cos\left(2\varphi \right)\right)\cdot {\vec{i}}_{g}+\\ +\omega \left(-L_{1}\cdot \sin\left(\varphi -\alpha \right)+2x_{CM2}\cos\left(2\varphi \right)-2y_{CM2}\sin\left(2\varphi \right)\right)\cdot {\vec{j}}_{g} \end{array},$$

(14) $$\begin{array}{l} {\vec{a}}_{CM2}=\omega^{2}\left(L_{1}\cdot \sin\left(\varphi -\alpha \right)-4x_{CM2}\cos\left(2\varphi \right)+4y_{CM2}\sin\left(2\varphi \right)\right)\cdot {\vec{i}}_{g}+\\ +\omega^{2}\left(-L_{1}\cdot \cos\left(\varphi -\alpha \right)-4x_{CM2}\sin\left(2\varphi \right)-4y_{CM2}\cos\left(2\varphi \right)\right)\cdot {\vec{j}}_{g} \end{array},$$

**Figure 10:**
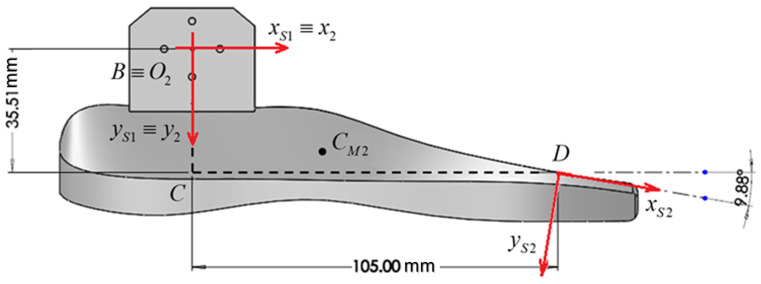
The coordinates of the positions where the accelerometers were attached.

Figure 10 and Table 7 show the coordinate systems used for the sole. The system $\left(x_{2},y_{2}\right)$ is the system bound to the sole, and the coordinate systems $\left(x_{s1},y_{s1}\right)$ and $\left(x_{s2},y_{s2}\right)$ are the coordinate systems of the sole-bound accelerometers, which have their origins at the acceleration measurement points.

Accelerometer measurement directions:

- In position 1, they are overlapped with the axes of the coordinate system linked to the sole;
- In position 2, the measurement directions are rotated by 9.88° trigonometrically.

Substituting the moving vertices of the coordinate system related to the base into the acceleration relation of point B results in the following expression:

(15) $${\vec{a}}_{B}=-L_{1}\cdot \omega_{1}^{2}\cdot \sin\left(\varphi +\alpha \right)\cdot {\vec{i}}_{2}-L_{1}\cdot \omega_{1}^{2}\cdot \cos\left(\varphi +\alpha \right)\cdot {\vec{j}}_{2},$$

This expression could also be found through directly writing the acceleration with respect to the system connected to the sole, knowing the direction (AB) and the module $\left(L_{1}\cdot \omega_{1}^{2}\right)$.

This expression is necessary for the interpretation of the experimental results, as it is also valid for the coordinate system of sensor 1.

For point D, which is the second position of the accelerometer, the relationships are similar to those of the center of mass. The position vector, velocity, and acceleration relative to the fixed system are given by:

(16) $$\begin{array}{l} {\vec{r}}_{D}=\vec{AB}+\vec{BC}+\vec{CD}=\\ =\left[-L_{1}\cdot \sin\left(\varphi -\alpha \right)+x_{D}\cdot \cos\left(2\varphi \right)-y_{D}\cdot \sin\left(2\varphi \right)\right]{\vec{i}}_{g}+\\ +\left[L_{1}\cdot \cos\left(\varphi -\alpha \right)+x_{D}\cdot \sin\left(2\varphi \right)+y_{D}\cdot \cos\left(2\varphi \right)\right]{\vec{j}}_{g} \end{array},$$

(17) $$\begin{array}{l} {\vec{v}}_{D}=\omega \left[-L_{1}\cdot \cos\left(\varphi -\alpha \right)-2x_{D}\cdot \sin\left(2\varphi \right)-2y_{D}\cdot \cos\left(2\varphi \right)\right]{\vec{i}}_{g}+\\ +\omega \left[-L_{1}\cdot \sin\left(\varphi -\alpha \right)+2x_{D}\cdot \cos\left(2\varphi \right)-2y_{D}\cdot \sin\left(2\varphi \right)\right]{\vec{j}}_{g} \end{array},$$

(18) $$\begin{array}{l} {\vec{a}}_{D}=\omega^{2}\left[L_{1}\cdot \sin\left(\varphi -\alpha \right)-4x_{D}\cdot \cos\left(2\varphi \right)+4y_{D}\cdot \sin\left(2\varphi \right)\right]{\vec{i}}_{g}+\\ +\omega^{2}\left[-L_{1}\cdot \cos\left(\varphi -\alpha \right)-4x_{D}\cdot \sin\left(2\varphi \right)-4y_{D}\cdot \cos\left(2\varphi \right)\right]{\vec{j}}_{g} \end{array},$$

With respect to the local system of the sole, using relations (10) and (11), it follows that:

(19) $${\vec{i}}_{g}={\vec{i}}_{2}\cdot \cos\left(2\varphi \right)-{\vec{j}}_{2}\cdot \sin\left(2\varphi \right),$$

(20) $${\vec{j}}_{g}={\vec{i}}_{2}\cdot \sin\left(2\varphi \right)+{\vec{j}}_{2}\cdot \cos\left(2\varphi \right),$$

and the expression for the acceleration of point *D* relative to the system attached to the sole is:

(21) $$\vec{a_{D}}=\omega^{2}\left[-L_{1}\cdot \sin\left(\varphi +\alpha \right)-4x_{D}\right]{\vec{i}}_{2}+\omega^{2}\left[-L_{1}\cdot \cos\left(\varphi +\alpha \right)-4y_{D}\right]{\vec{j}}_{2}.$$

This relationship can also be obtained from Euler’s formula written for the sole:

(22) $$\vec{a_{D}}=\vec{a_{B}}+\vec{a_{DB_{n}}}+\vec{a_{DB_{t}}},$$

where $\vec{a_{CDB_{n}}}=\vec{\omega_{2}}\times \left(\vec{\omega_{2}}\times \vec{BD}\right)$ has the direction *BD*, with the orientation from *D* to *B* and the module $BD\cdot \omega_{2}^{2}=\sqrt{x_{2D}^{2}+y_{2D}^{2}}\cdot \omega^{2}$, and $\vec{a_{DB_{t}}}=\vec{\varepsilon_{2}}\times \vec{BD}=0$, because $\omega_{2}=ct$.

Compared to the sensor-related system $\left(x_{s2},y_{s2}\right)$, which is rotated relative to the system $\left(x_{2},y_{2}\right)$ with the angle β=9.88°, the acceleration becomes

(23) $$\begin{array}{l} \vec{a_{D}}=\omega^{2}\left[-L_{1}\cdot \sin\left(\varphi +\alpha +\beta \right)-4x_{2D}\cdot \cos\left(\beta \right)-4y_{2D}\cdot \sin\left(\beta \right)\right]{\vec{i}}_{s2}+\\ +\omega^{2}\left[-L_{1}\cdot \cos\left(\varphi +\alpha +\beta \right)+4x_{2D}\cdot \sin\left(\beta \right)-4y_{2D}\cdot \cos\left(\beta \right)\right]{\vec{j}}_{s2} \end{array}.$$

In order to highlight the movement described by the above laws, three-way accelerometers (PCB Piezotronics 356A43 S/N LW348378) were used. Accelerometers were attached to the foot in two positions: on the sole, and on the axis of the calf–foot joint.

#### 2.3.2. Dynamics

**Figure 11:**
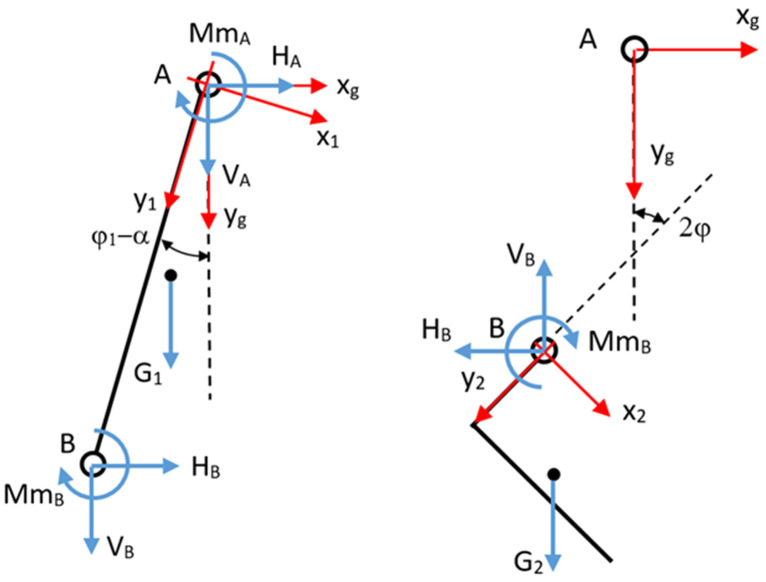
The calf and sole insulation.

To determine the forces in the joints, we isolate the system (Figure 11). For each body, we write the momentum theorem with respect to the fixed reference system, and the kinetic momentum theorem with respect to the center of mass.

For the calf (body 1), we obtain the following relations:

(24) $$M_{1}a_{CM1_{xg}}=H_{A}+H_{B},$$

(25) $$M_{1}a_{CM1_{yg}}=V_{A}+V_{B}+G_{1},$$

(26) $$\begin{array}{l} \frac{d}{dt}\left(K_{CM1_{z1}}\right)=0=M_{CM1}{\left(\vec{H_{A}}\right)}_{z1}+M_{CM1}{\left(\vec{V_{A}}\right)}_{z1}+M_{CM1}{\left(\vec{H_{B}}\right)}_{z1}+\\ +M_{CM1}{\left(\vec{V_{B}}\right)}_{z1}+Mm_{A}+Mm_{B} \end{array},$$

where $M_{1}$ is the mass of the calf, $G_{1}$ is the weight of the calf, $a_{CM1_{xg}}$ and $a_{CM1_{yg}}$ are the components of the acceleration of the center of mass (relation (8)), and $Mm_{A}$ and $Mm_{B}$ are the motor moments in the joints, which produce the movement.

The moment about the center of mass of the force $H_{A}$ is determined via

(27) $$\vec{M_{CM1}}\left(\vec{H_{A}}\right)=\vec{C_{M1}A}\times \vec{H_{A}}=\left|\begin{array}{ccc} {\vec{i}}_{1} & {\vec{j}}_{1} & {\vec{k}}_{1}\\ x1_{A}-x1_{CM1} & y1_{A}-y1_{CM1} & 0\\ H_{A_{x1}} & H_{A_{y1}} & 0 \end{array}\right|,$$

with the component along the Oz axis being

(28) $$\begin{array}{l} M_{CM1}{\left(\vec{H_{A}}\right)}_{z1}=\left(x1_{A}-x1_{CM1}\right)H_{A_{y1}}-\left(y1_{A}-y1_{CM1}\right)H_{A_{x1}}=\\ =y1_{CM1}H_{A_{x1}}-x1_{CM1}H_{A_{y1}}=H_{A}\left(y1_{CM1}\cos\left(\varphi -\alpha \right)+x1_{CM1}\sin\left(\varphi -\alpha \right)\right) \end{array},$$

Similarly, the components of the other moments result in the following:

(29) $$\begin{array}{l} M_{CM1}{\left(\vec{V_{A}}\right)}_{z1}=y1_{CM1}V_{A_{x1}}-x1_{CM1}V_{A_{y1}}=\\ =V_{A}\left(y1_{CM1}\sin\left(\varphi -\alpha \right)-x1_{CM1}\cos\left(\varphi -\alpha \right)\right) \end{array},$$

(30) $$\begin{array}{l} M_{CM1}{\left(\vec{H_{B}}\right)}_{z1}=\left(x1_{B}-x1_{CM1}\right)H_{B_{y1}}-\left(y1_{B}-y1_{CM1}\right)H_{B_{x1}}=\\ =H_{B}\left(\left(y1_{CM1}-y1_{B}\right)\cos\left(\varphi -\alpha \right)+x1_{CM1}\sin\left(\varphi -\alpha \right)\right) \end{array},$$

(31) $$\begin{array}{l} M_{CM1}{\left(\vec{V_{B}}\right)}_{z1}=\left(x1_{B}-x1_{CM1}\right)V_{B_{y1}}-\left(y1_{B}-y1_{CM1}\right)V_{B_{x1}}=\\ =V_{B}\left(\left(y1_{CM1}-y1_{B}\right)\sin\left(\varphi -\alpha \right)-x1_{CM1}\cos\left(\varphi -\alpha \right)\right) \end{array},$$

For the calf (body 2), we obtain the following relations:

(32) $$M_{2}a_{CM2_{xg}}=-H_{B},$$

(33) $$M_{2}a_{CM2_{yg}}=-V_{B}+G_{2},$$

(34) $$\frac{d}{dt}\left(K_{CM2_{z2}}\right)=0=M_{CM2}{\left(-\vec{H_{B}}\right)}_{z2}+M_{CM2}{\left(-\vec{V_{B}}\right)}_{z2}-Mm_{B},$$

where $M_{2}$ is the mass of the calf, $G_{2}$ is the weight of the calf, and $a_{CM2_{xg}}$ and $a_{CM2_{yg}}$ are the components of the acceleration of the center of mass (relation (14)).

From relations (32) and (33), we obtain the force components in joint *B*, $H_{B}$, and $V_{B}$:

(35) $$\begin{array}{l} H_{B}\left(\varphi \right)=-M_{2}a_{CM2_{x}}=\\ =-M_{2}\omega^{2}\left(L_{1}\cdot \sin\left(\varphi -\alpha \right)-4x_{CM2}\cos\left(2\varphi \right)+4y_{CM2}\sin\left(2\varphi \right)\right) \end{array},$$

(36) $$\begin{array}{l} V_{B}=-M_{2}a_{CM2_{y}}+G_{2}=\\ =-M_{2}\omega^{2}\left(-L_{1}\cdot \cos\left(\varphi -\alpha \right)-4x_{CM2}\sin\left(2\varphi \right)-4y_{CM2}\cos\left(2\varphi \right)\right)+G_{2} \end{array},$$

and from relation (34), the motor moment in joint *B* is given by

(37) $$\begin{array}{l} Mm_{B}\left(\varphi \right)=M_{CM2}{\left(-\vec{H_{B}}\right)}_{z}+M_{CM2}{\left(-\vec{V_{B}}\right)}_{z}=\\ =H_{B}\left(\varphi \right)\left(-y2_{CM2}\cos\left(2\varphi \right)-x2_{CM2}\sin\left(2\varphi \right)\right)+\\ +V_{B}\left(\varphi \right)\left(y2_{CM2}\sin\left(2\varphi \right)-x2_{CM2}\cos\left(2\varphi \right)\right) \end{array},$$

From relations (24)–(26), we obtain the components of the connecting force in joint A, and the motor moment from this joint:

(38) $$\begin{array}{l} H_{A}\left(\varphi \right)=M_{1}a_{CM1_{xg}}-H_{B}=M_{1}a_{CM1_{xg}}+M_{2}a_{CM2_{xg}}\\ =M_{1}\omega^{2}\left(-x_{CM1}\cos\left(\varphi -\alpha \right)+y_{CM1}\sin\left(\varphi -\alpha \right)\right)+\\ +M_{2}\omega^{2}\left(L_{1}\cdot \sin\left(\varphi -\alpha \right)-4x_{CM2}\cos\left(2\varphi \right)+4y_{CM2}\sin\left(2\varphi \right)\right) \end{array},$$

(39) $$\begin{array}{l} V_{A}\left(\varphi \right)=M_{1}a_{CM1_{yg}}-V_{B}-G_{1}=\\ =M_{1}\omega^{2}\left(-x_{CM1}\sin\left(\varphi -\alpha \right)-y_{CM1}\cos\left(\varphi -\alpha \right)\right)+\\ +M_{2}\omega^{2}\left(-L_{1}\cdot \cos\left(\varphi -\alpha \right)-4x_{CM2}\sin\left(2\varphi \right)-4y_{CM2}\cos\left(2\varphi \right)\right)-\\ -G_{1}-G_{2} \end{array},$$

(40) $$\begin{array}{l} Mm_{A}\left(\varphi \right)=-M_{CM1}{\left(\vec{H_{A}}\right)}_{z1}-M_{CM1}{\left(\vec{V_{A}}\right)}_{z1}-M_{CM1}{\left(\vec{H_{B}}\right)}_{z1}-\\ -M_{CM1}{\left(\vec{V_{B}}\right)}_{z1}-Mm_{B}=\\ =-H_{A}\left(\varphi \right)\left(y1_{CM1}\cos\left(\varphi -\alpha \right)+x1_{CM1}\sin\left(\varphi -\alpha \right)\right)-\\ -V_{A}\left(\varphi \right)\left(y1_{CM1}\sin\left(\varphi -\alpha \right)-x1_{CM1}\cos\left(\varphi -\alpha \right)\right)-\\ -H_{B}\left(\varphi \right)\left(\left(y1_{CM1}-y1_{B}\right)\cos\left(\varphi -\alpha \right)+x1_{CM1}\sin\left(\varphi -\alpha \right)\right)-\\ -V_{B}\left(\varphi \right)\left(\left(y1_{CM1}-y1_{B}\right)\sin\left(\varphi -\alpha \right)-x1_{CM1}\cos\left(\varphi -\alpha \right)\right)-\\ -H_{B}\left(\varphi \right)\left(-y2_{CM2}\cos\left(2\varphi \right)-x2_{CM2}\sin\left(2\varphi \right)\right)-\\ -V_{B}\left(\varphi \right)\left(y2_{CM2}\sin\left(2\varphi \right)-x2_{CM2}\cos\left(2\varphi \right)\right) \end{array},$$

#### 2.3.3. Equipment Used in the Determination of Mechanical Characteristics

**Figure 12:**
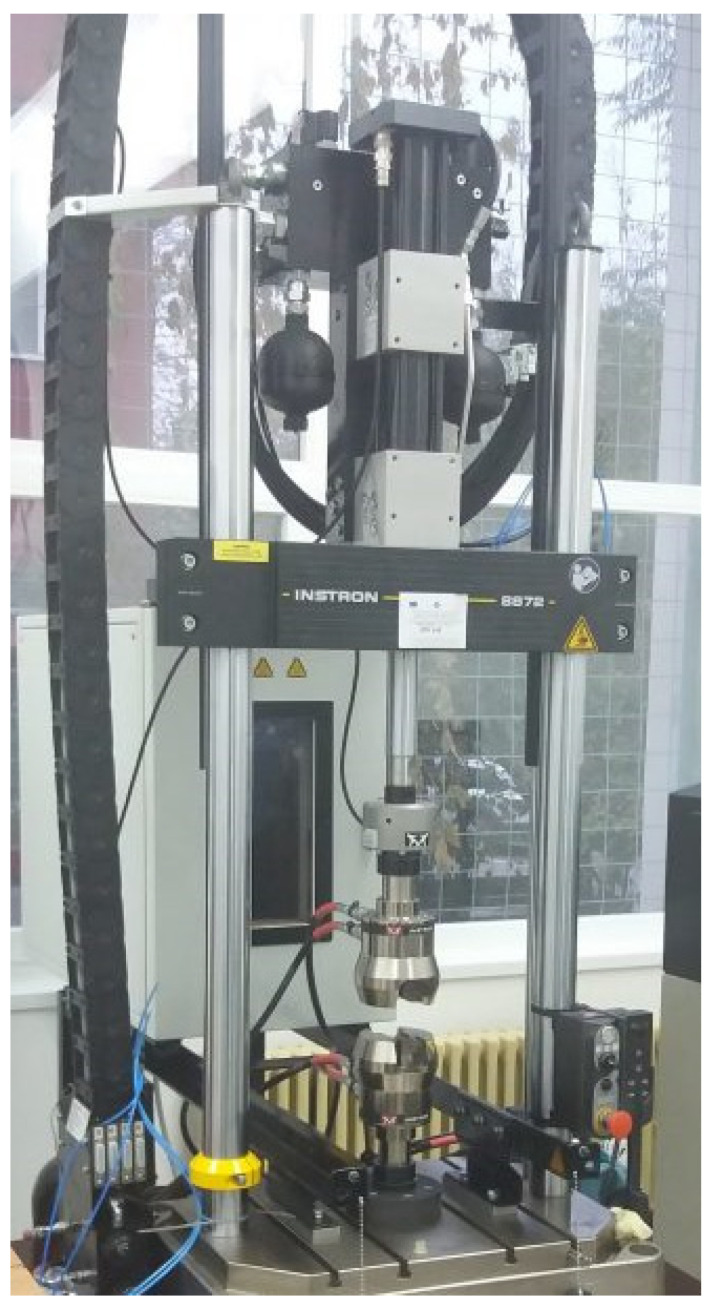
INSTRON 8872 universal testing machine [30].

**Figure 13:**
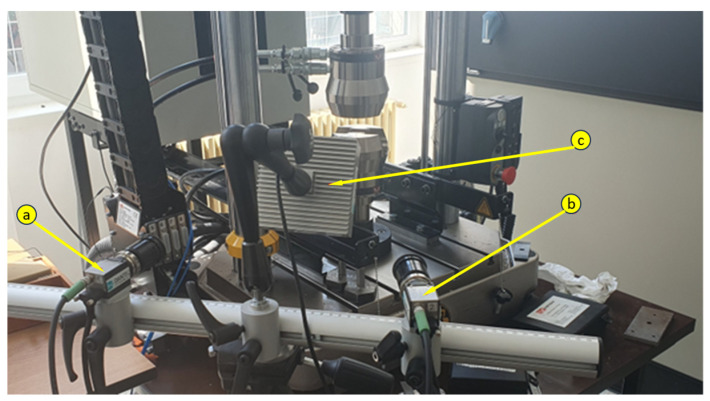
Image correlation system (a—Camera 1, b—Camera 2, c—light source).

In order to determine the mechanical characteristics, two pieces of equipment were used; namely, the INSTRON 8872 universal testing machine (Figure 12), and the Dantec image correlation system (Figure 13). All the mechanical tests were performed at 1 mm/min.

The characteristics of the INSTRON 8872 are shown in Table 8.

The specifications of the image correlation system are shown in Table 9.

**Figure 14:**
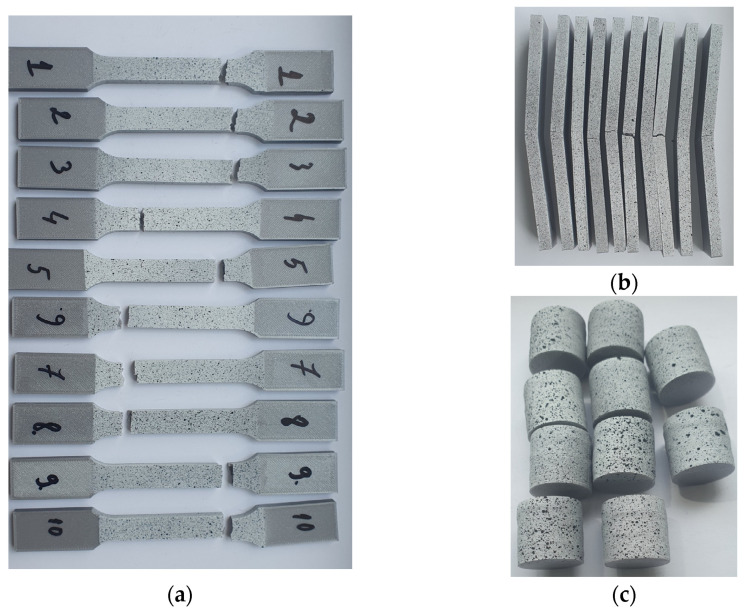
Tested specimens: (**a**) traction, (**b**) bending, (**c**) compression.

Figure 14 shows the tested samples.

The specific deformations were taken from the application of the image correlation method, and the values of the normal stresses appearing during the tensile and compressive stresses were determined via the following relationship:

(41) $$\sigma =\frac{F}{A},$$

where *F* is the axial force applied to the specimen; and *A* is the cross-sectional area of the specimen.

For the three-point bending test, the formulas used to calculate the normal stress and the specific strain were as follows:

(42) $$\sigma =\frac{1.5\cdot s}{b\cdot h^{2}},$$

(43) $$\varepsilon =\frac{d\cdot h\cdot 6}{s^{2}},$$

where *s* is the distance between the supports (support span); *d* is the sample arrow; *b* is the width of the sample; and *h* is the thickness of the specimen.

#### 2.3.4. Simulation via the Finite Element Method

ANSYS software was used for finite element modeling. The modeling was performed for a transient regime, using the Transient Structural module.

The contacts used between these components are as follows:

- For the sole:
  o A “frictionless” type for the contact between the foot and the clamping bush with the servomotor;
  o A “bound” type for the rest of the contacts;
- For the calf:
  o A “frictionless” type for the contact between the calf and the clamping bush with the servomotor in the knee, and for the contact between the foot actuation servomotor and the calf;
  o A “bound” type for the rest of the contacts;
- For the knee:
  o A “frictionless” type for the contact between the knee and the calf actuation servomotor;
  o A “bound” type for the rest of the contacts.

Between the sole and the calf, and between the calf and the knee, respectively, “revolute” connections were defined between the shafts of the actuators and the connecting bushings. This type of link allows the imposition of boundary conditions on angular displacements, angular velocities, or angular accelerations.

**Figure 15:**
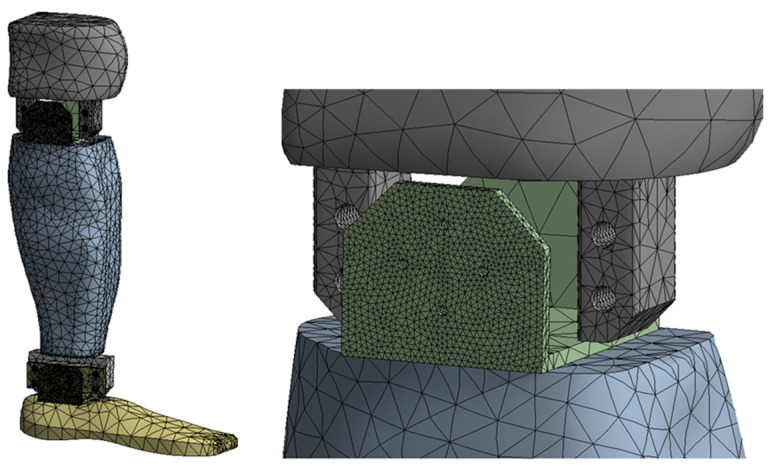
Finite element discretization.

The finite element discretization, and a detail of the discretization in the knee area, are shown in Figure 15. An average element size of 12 mm was used for the discretization, with refinement on contact surfaces with an average size of 1 mm, resulting in 120,592 nodes and 69,622 elements.

**Figure 16:**
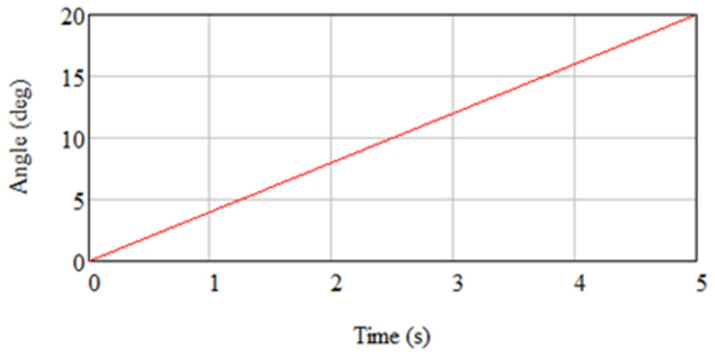
“Revolved” connections with the imposition of angular displacements.

Zero displacements were considered on the upper surface of the knee. In the “revolute”-type links, angular displacements were imposed, according to the law presented in Figure 16.

**Figure 17:**
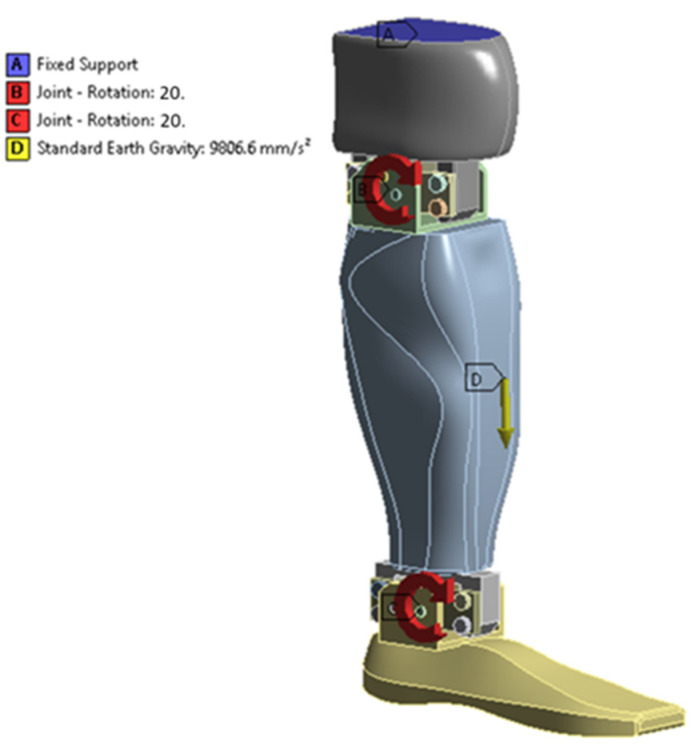
The boundary conditions, gravitational acceleration, and rotations imposed in the joints on the geometric configuration from the initial time instant.

The mass forces due to the user’s weight were also taken into account, defining the gravitational acceleration (Figure 17).

The analysis was transient, with a time interval of $\left[0;5\right]$ seconds, and a minimum time step of $10^{-4}\;s$. The displacements were considered large, and the results were saved at each time step.

## 3. Results

### 3.1. Experimental Measurements

To allow the realization of the movements of the leg prosthesis, it was equipped with hardware, as follows: Raspberry Pi 4, two servo motors, a motor driver module, and an external power supply (this variant being sufficient to demonstrate the concept of the neural control of the prosthesis) and, for controlling the prosthesis with the power of the mind, an EMOTIV Insight headset (Figure 1) was used (this being responsible for capturing electrical signals from the brain, and converting them into specific commands, which could later be used to control the bionic lower limb prosthesis). The testing of the implemented system was carried out with a healthy male participant, who used the neural helmet to train two commands necessary for the movement of the leg prosthesis. Compared to other advanced headsets that contain many electrodes, the performance of the system is relatively good in terms of the EEG signal obtained from the EMOTIV Insight Neuronal Headset, because it provides the desired decoding and processing for the brain signals.

**Figure 18:**
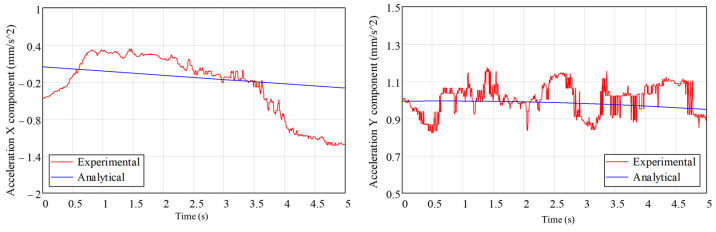
The time variation in the experimentally and analytically determined acceleration components for sensor 1.

**Figure 19:**
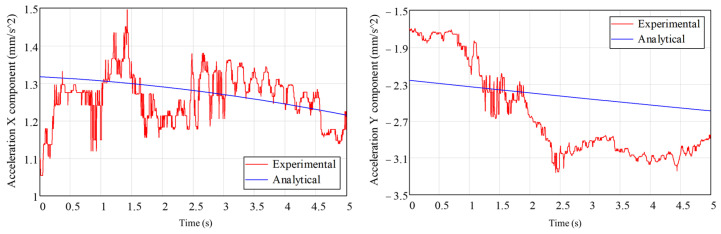
The time variation in experimentally and analytically determined acceleration components for sensor 2.

Figure 18 and Figure 19 show the experimentally determined acceleration components, with an acquisition frequency of 300 Hz, for the two accelerometers. Analytically determined acceleration variations are also presented on the same graphs.

### 3.2. Mechanical Test Results

**Figure 20:**
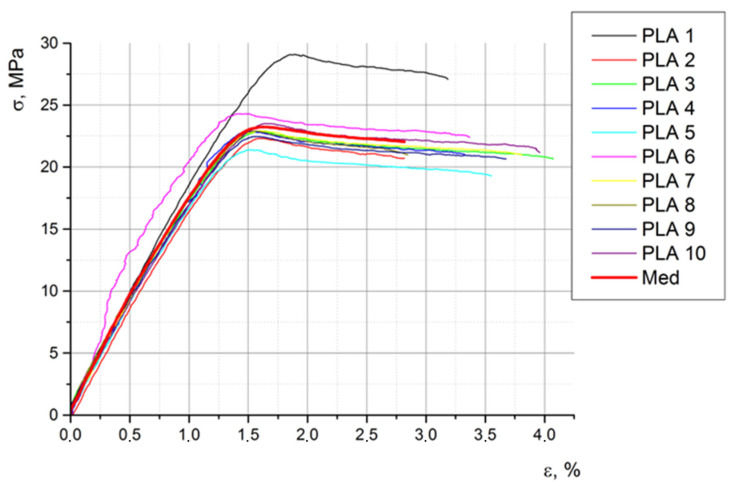
The stress–strain curves of the ten specimens subjected to traction.

**Figure 21:**
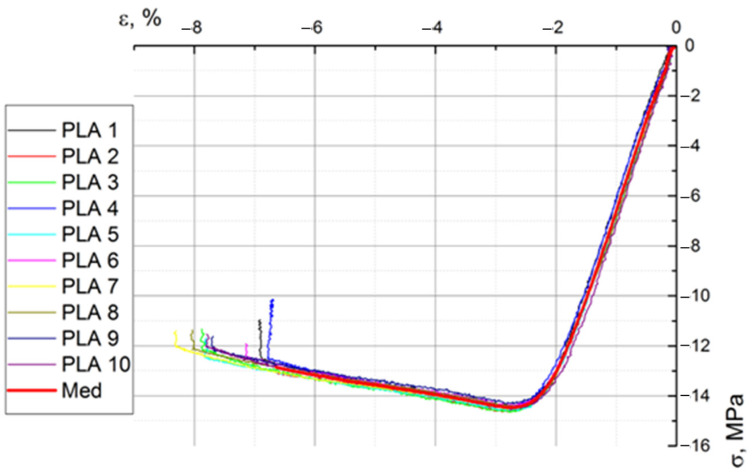
The stress–strain curves of the ten specimens subjected to compression.

**Figure 22:**
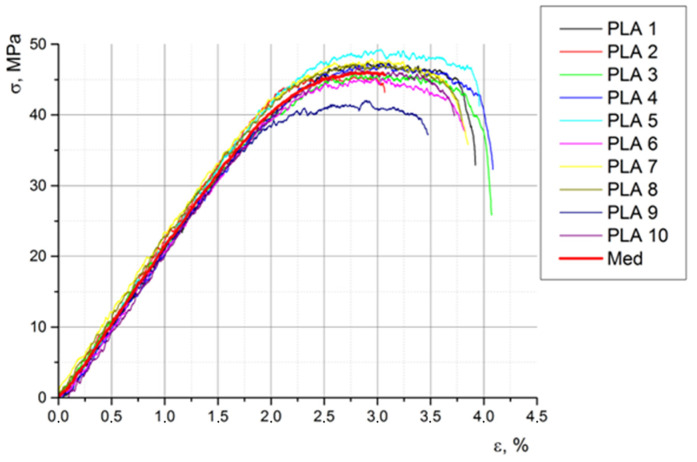
The stress–strain curves of the ten specimens subjected to bending.

Figure 20, Figure 21 and Figure 22 show the characteristic curves for all ten specimens subjected to tension, compression, and bending, respectively.

Table 10 shows the main mechanical properties obtained via the tensile test.

Following the compression test, a series of mechanical characteristics of PLA were obtained, which are presented in Table 11.

As a result of the bending tests, the following mechanical properties of PLA were obtained, as shown in Table 12.

Following the statistical analysis of the experimental results, we concluded that the average values, regarding the mechanical characteristics of the material from which the prosthesis was made (experimental model), were sufficient for the present study. In further development, we will perform a statistical analysis regarding the behavior of the bionic prosthesis in response to the different (real) demands that will be identified as reasonable in the simulation of bipedal walking.

### 3.3. The Results Obtained from the Analysis via the Finite Element Method

**Figure 23:**
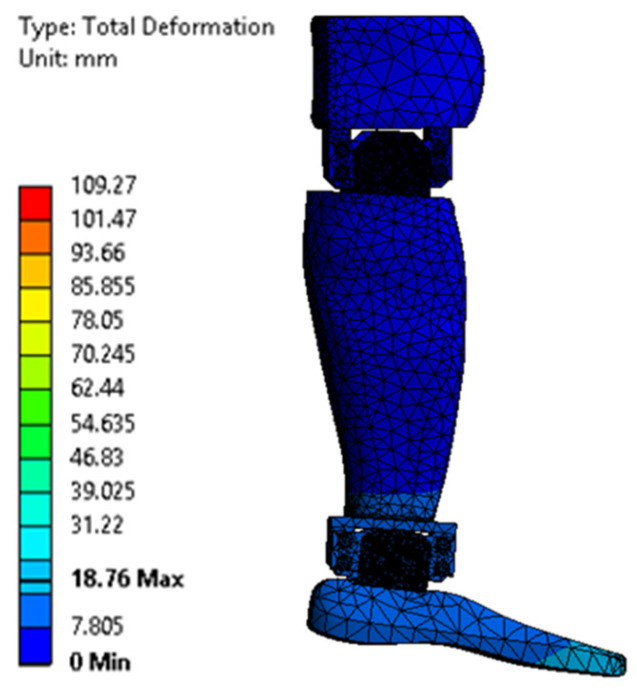
Total displacement at 0.833 s.

**Figure 24:**
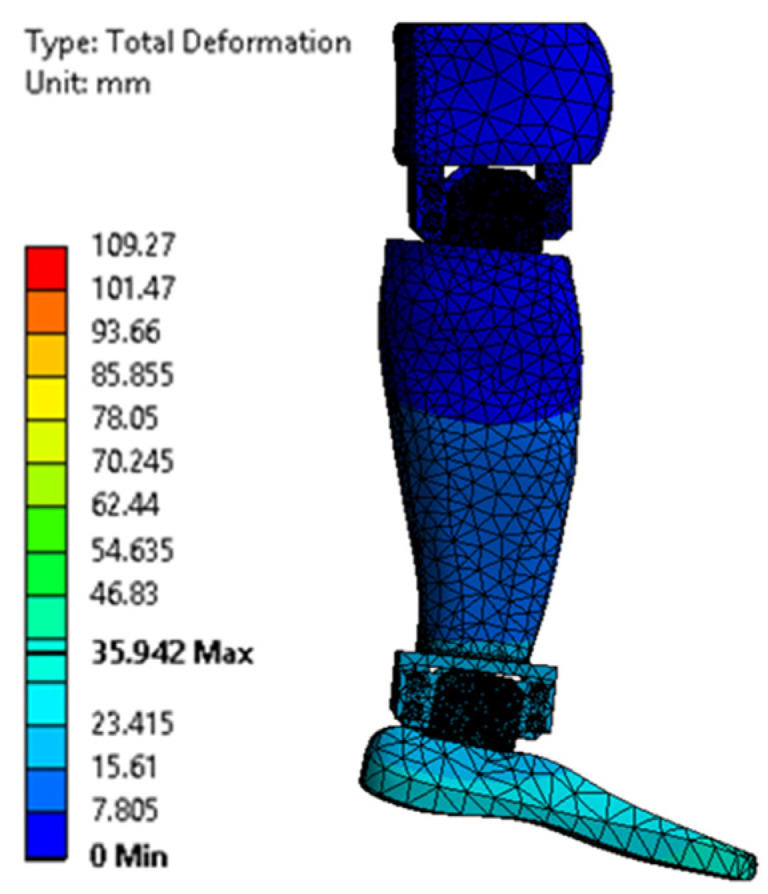
Total displacement at 1.667 s.

**Figure 25:**
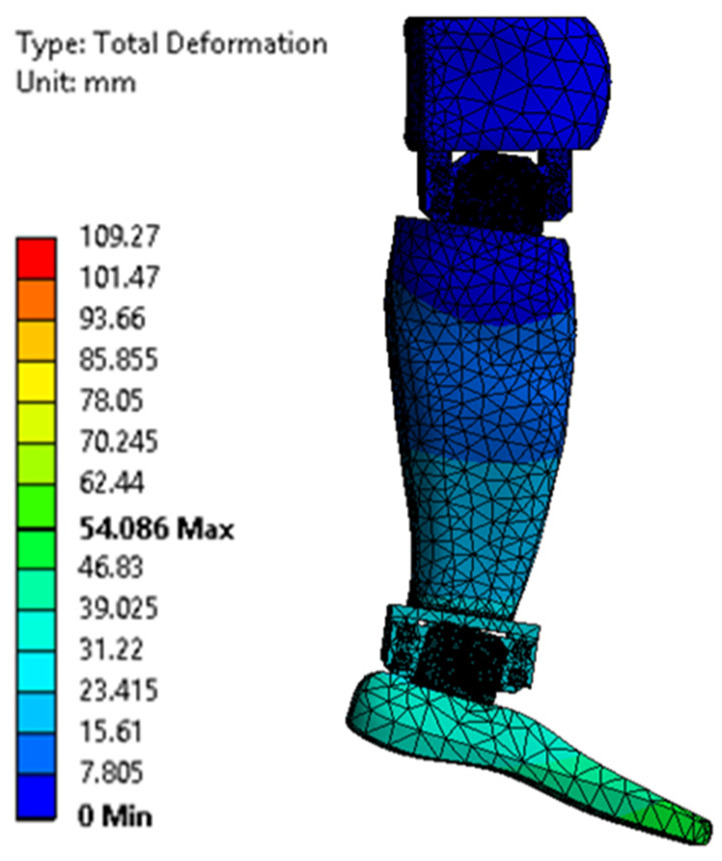
Total displacement at 2.5 s.

**Figure 26:**
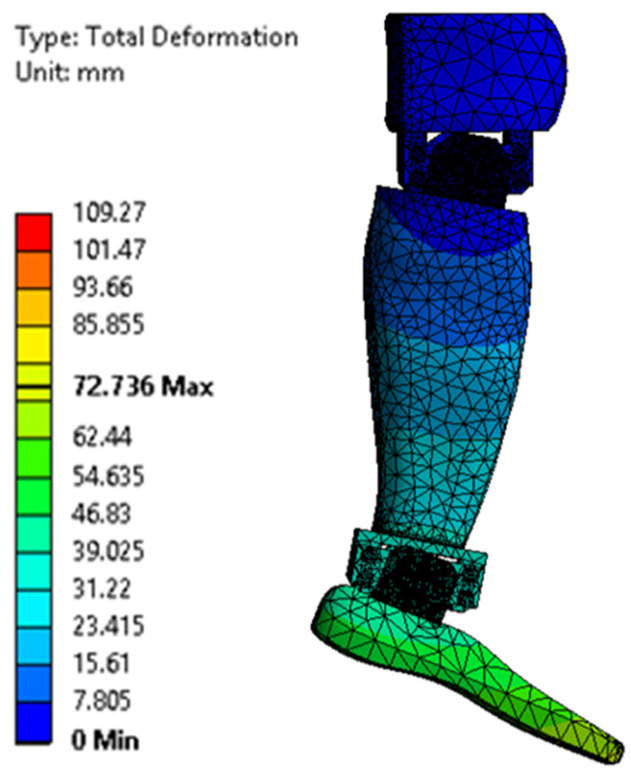
Total displacement at 3.333 s.

**Figure 27:**
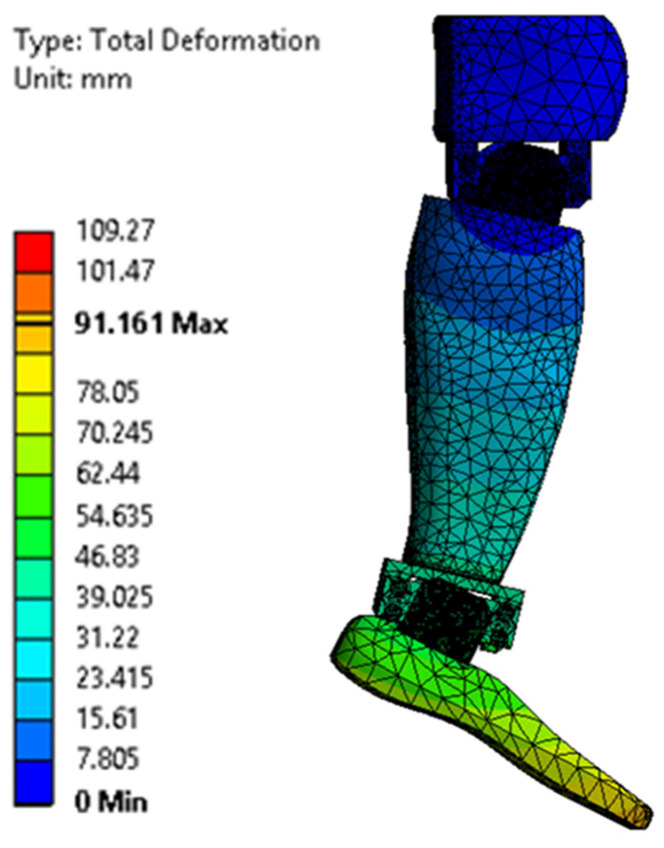
Total displacement at 4.167 s.

**Figure 28:**
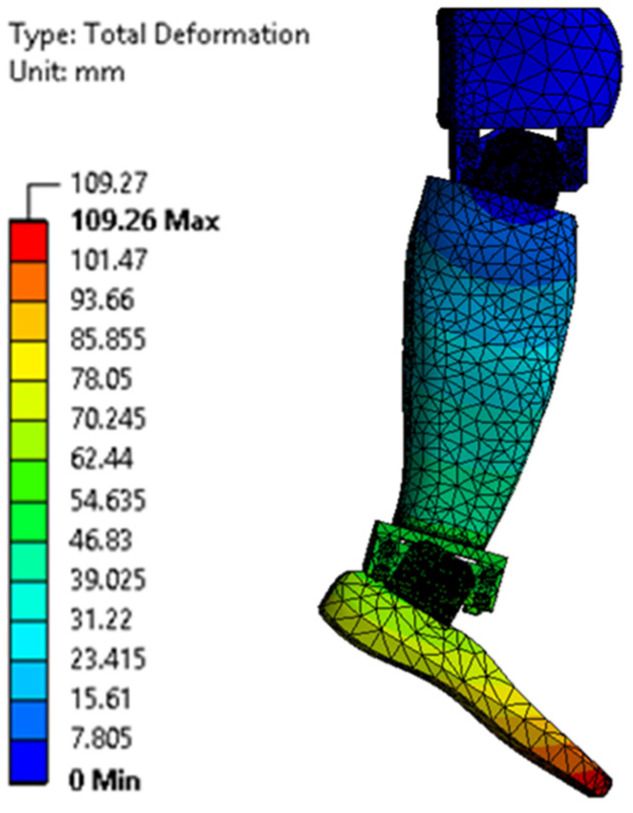
Total displacement at 5 s.

Figure 23, Figure 24, Figure 25, Figure 26, Figure 27 and Figure 28 show the resulting displacements in the components made of PLA, for the times of 0.833, 1.667, 2.5, 3.333, 4.167, and 5 s.

**Figure 29:**
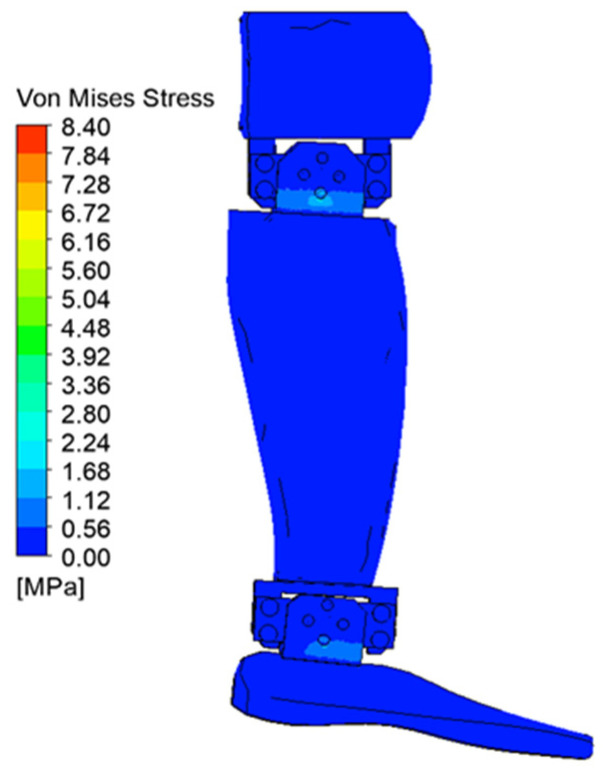
Von Mises stress at 0.833 s.

**Figure 30:**
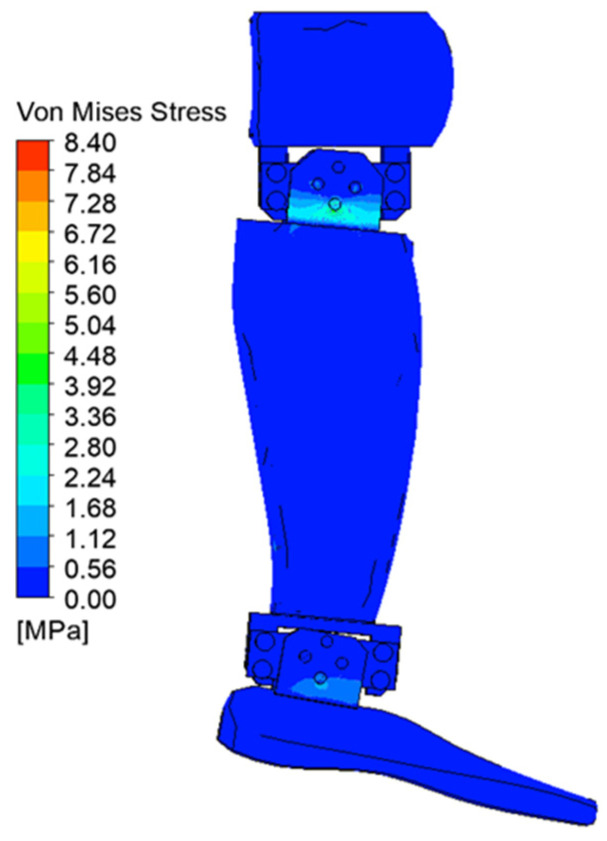
Von Mises stress at 1.667 s.

**Figure 31:**
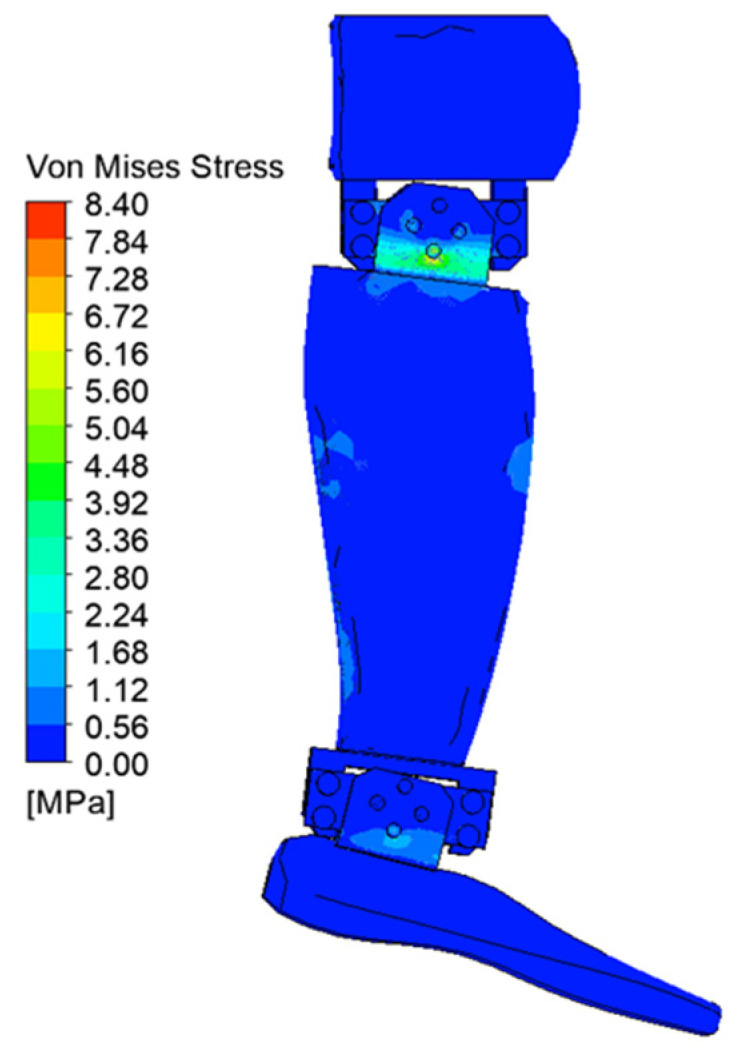
Von Mises stress at 2.5 s.

**Figure 32:**
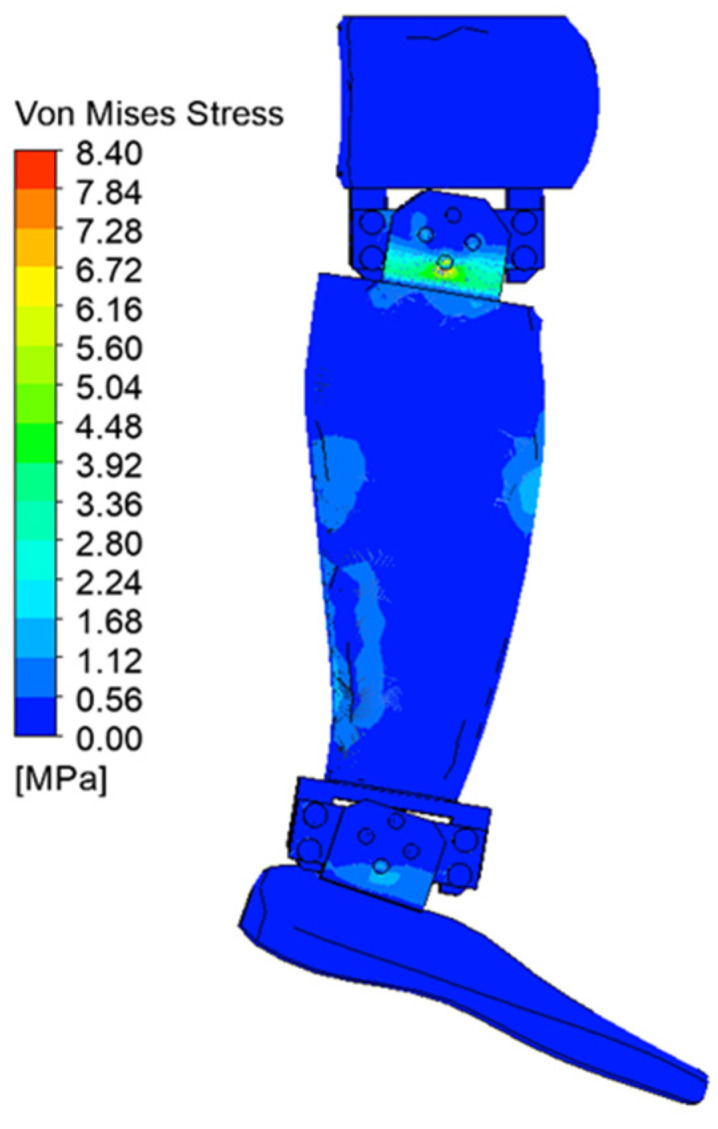
Von Mises stress at 3.333 s.

**Figure 33:**
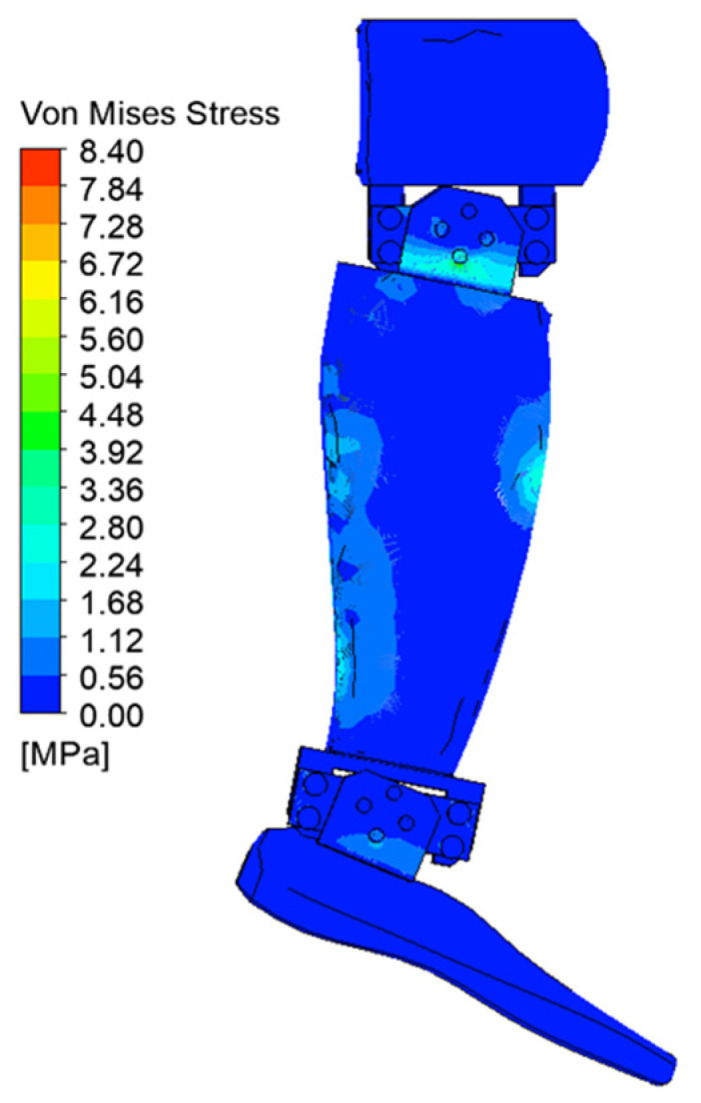
Von Mises stress at 4.167 s.

**Figure 34:**
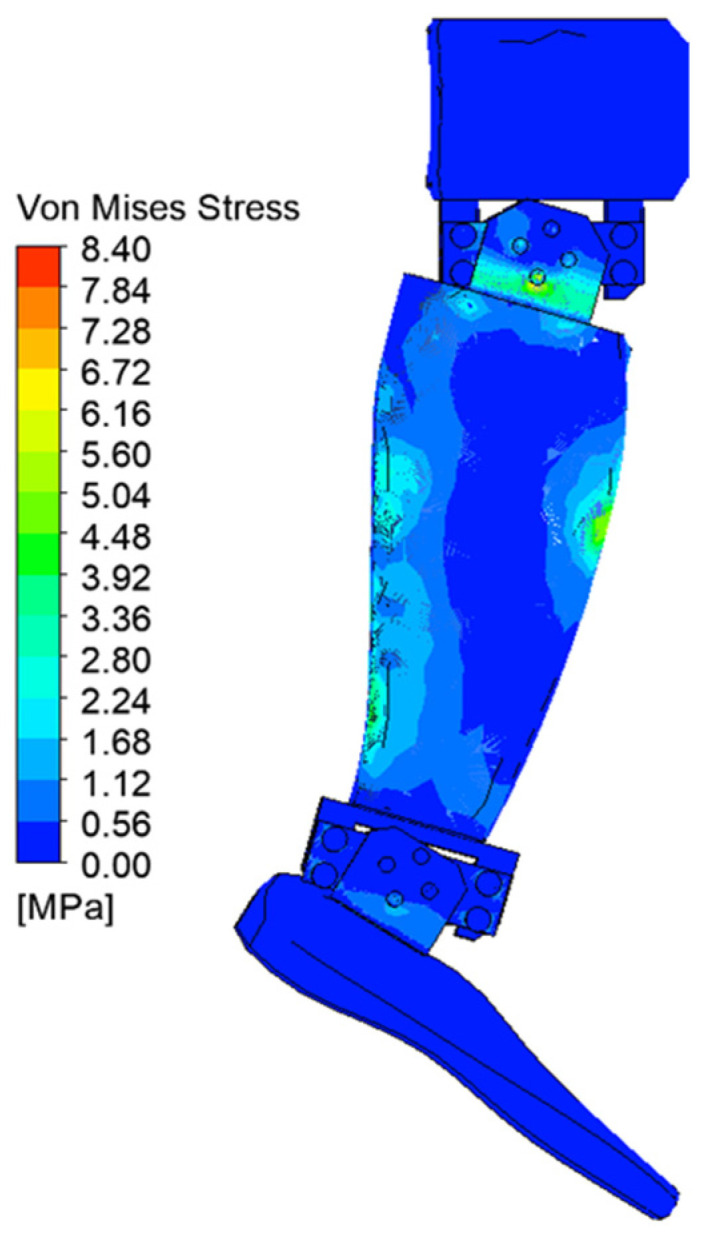
Von Mises stress at 5 s.

Figure 29, Figure 30, Figure 31, Figure 32, Figure 33 and Figure 34 show the equivalent stresses in the components made of PLA, for the times of 0.833, 1.667, 2.5, 3.333, 4.167, and 5 s.

**Figure 35:**
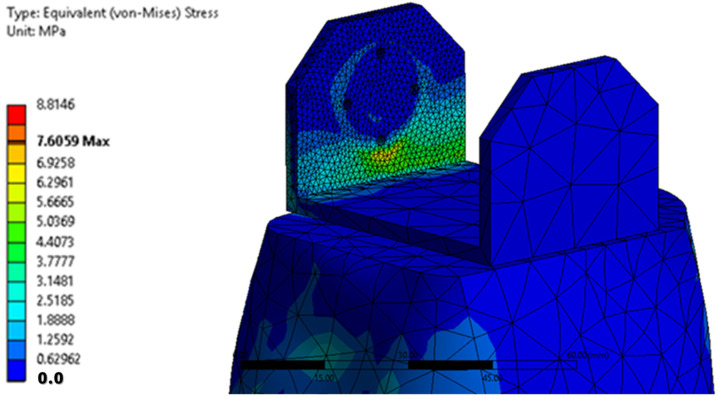
A detail of the maximum equivalent voltage area.

The maximum equivalent von Mises stress is reached when the angle of inclination of the calf to the vertical is maximum, i.e., 20°. This value is reached on the inside of the calf clamping yoke, in the area of contact with the servomotor clamping bush, in the knee joint. Figure 35 shows a detail of the maximum stress area. The ultimate stress is lower than the breaking strength of the PLA material.

**Figure 36:**
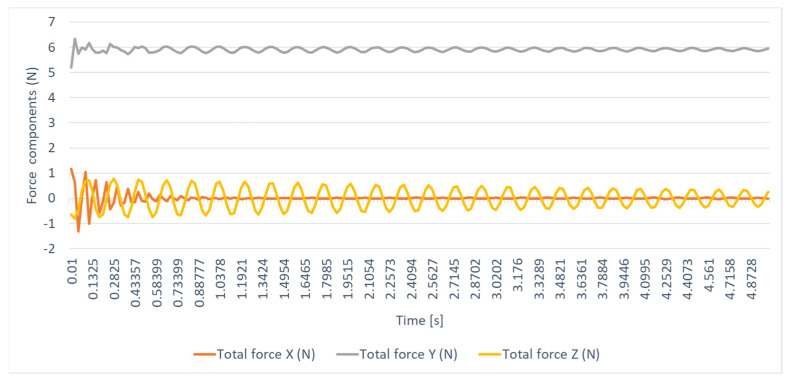
The time variation in the knee–joint bond force components.

**Figure 37:**
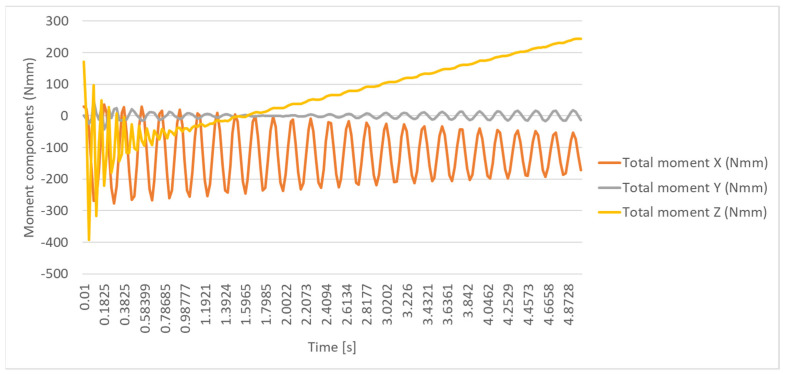
The time variation in the link moment components in the knee joint.

**Figure 38:**
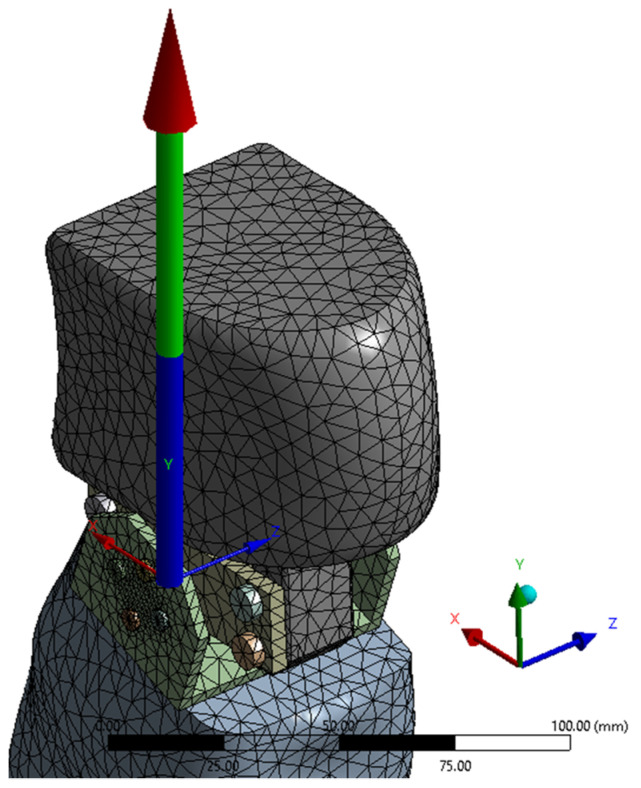
The resultant force.

**Figure 39:**
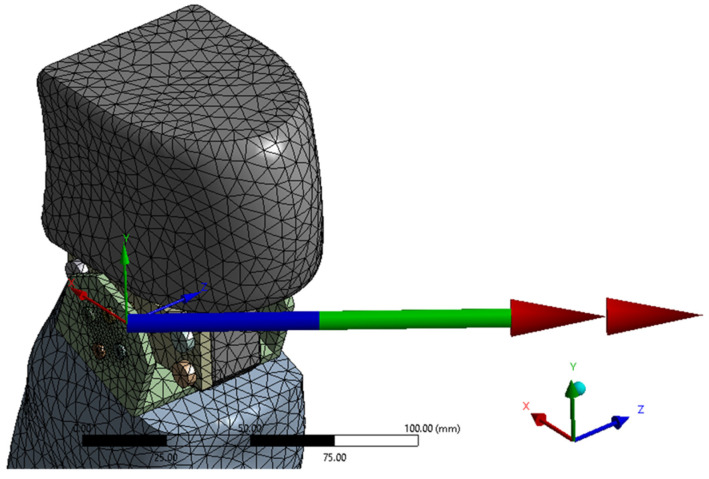
The resulting moment.

In the knee joint, the time variations in force and moment by components, expressed with respect to the local system, are presented in Figure 36, Figure 37, Figure 38 and Figure 39. The maximum stress is observed when the leg and sole are rotated by 20 degrees, with the moment in the joint reaching the maximum value of 717.05 N mm.

**Figure 40:**
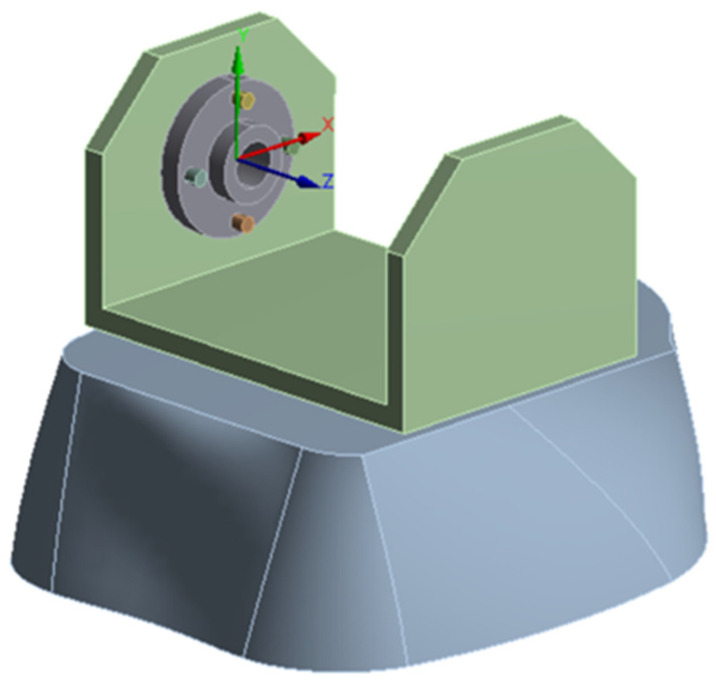
The simplified geometry of the knee joint.

For a more accurate calculation in the zone of maximum stresses, a simplified geometry was used (Figure 40, with the coordinate system of the knee joint), via the sectioning of the calf with a plane at a distance of 20 mm from the knee yoke.

**Figure 41:**
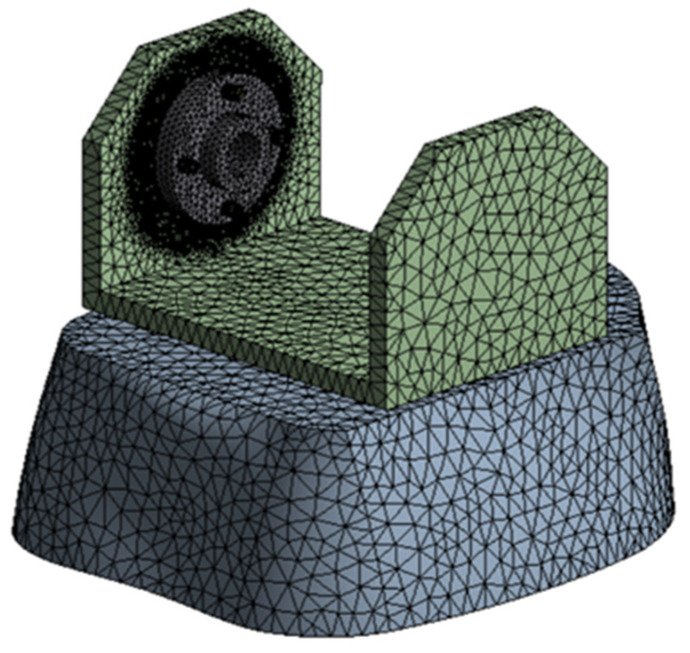
The discretization of the knee joint in the contact area between the actuator bushing and the knee joint.

The geometry was finely discretized in the contact area between the actuator bushing and the knee yoke, with 221,160 nodes and 135,931 elements (Figure 41).

“Frictionless” contacts were defined between the clamping screws and the knee yoke, and between the servomotor clamping bushing and the knee yoke, and “bound” contacts were defined between the screws and the clamping bushing. In the lower part of the structure, on the section plane, the nodes were considered blocked. The maximum force and moment determined in the transient analysis were imposed on the inner cylindrical surface of the clamping bush.

**Figure 42:**
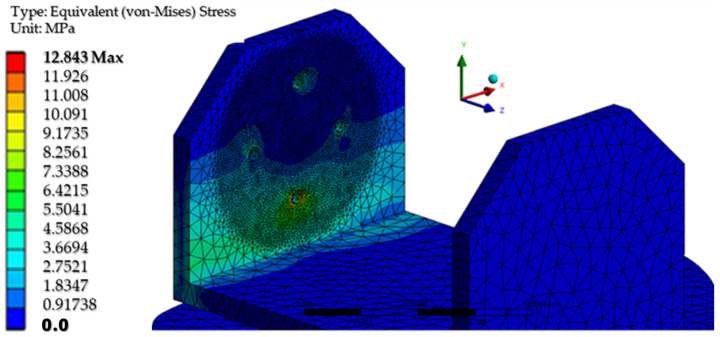
The equivalent von Mises stresses in PLA.

Figure 42 shows the von Mises equivalent stresses for the PLA component. A maximum stress of 12.84 MPa is noted in the vicinity of the screw hole at the bottom of the knee yoke. Below this area is the value of the voltage determined during the transient calculation.

**Figure 43:**
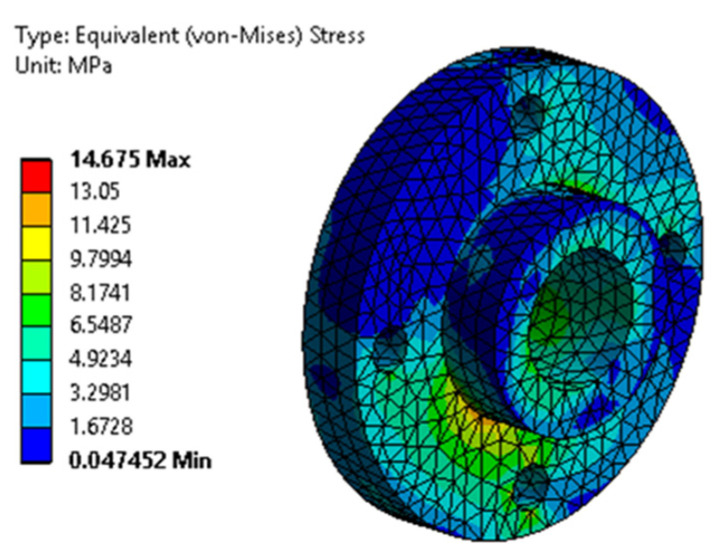
The von Mises equivalent stresses from the bushing with the servomotor.

**Figure 44:**
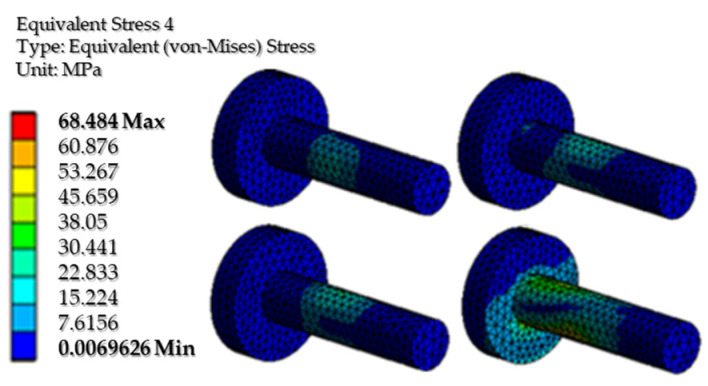
The von Mises equivalent stresses from the fastening screws.

Figure 43 and Figure 44 show the equivalent stresses in the servomotor mounting bushing, and the knee yoke mounting bolts, respectively. It is noted that the maximum stress is small compared to the yield stress of the two materials.

## 4. Discussion

It is noted both from the experimental results, and from the finite element model, that vibrations exist in the mechanical system when it is in motion. This aspect is due to the fact that, at the initial moment, the leg is in static equilibrium, and when the actuators act, the components must accelerate from zero rotational speed to the imposed speed of 0.069 rad/s. This action produces vibration in the absence of torque control. Another solution is to use a function for the displacement law that ensures acceleration and deceleration over a longer period of time, with an intermediate interval of constant speed. As vibrations cannot be eliminated, a future research direction would be to study the fatigue of PLA components.

From the analysis of Figure 18 and Figure 19, in which the components of the accelerations on the measurement directions of the accelerometers have been determined experimentally and analytically, a good agreement is noted. For better control of the position of the leg components, and to more easily compare experimental data with analytical or numerical data, it is necessary to use angular displacement sensors (encoders).

Another future direction of interest is the obstacle approach. In this case, the forces that apply to the leg structure can increase substantially. For this reason, we will introduce an elastic joint system.

**Figure 45:**
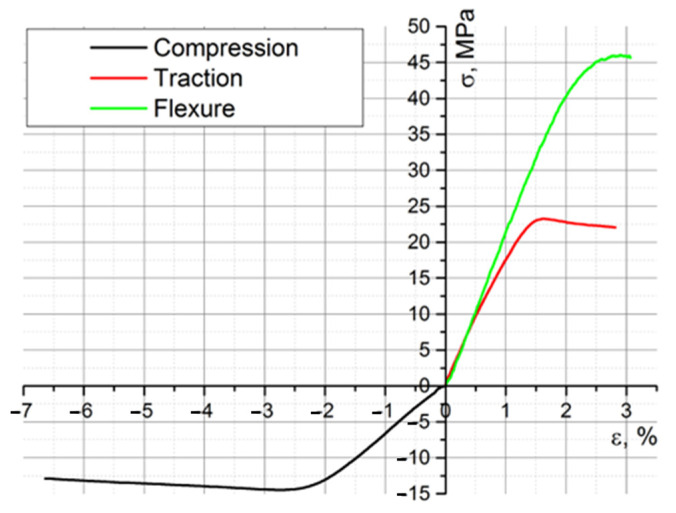
The characteristic curves of the three tests performed for PLA.

Regarding the elastic and mechanical characteristics of PLA, Figure 45 shows the values of the transverse contraction coefficient for the tensile and compression tests. The transverse shrinkage coefficient could not be obtained following the three-point bending test.

**Figure 46:**
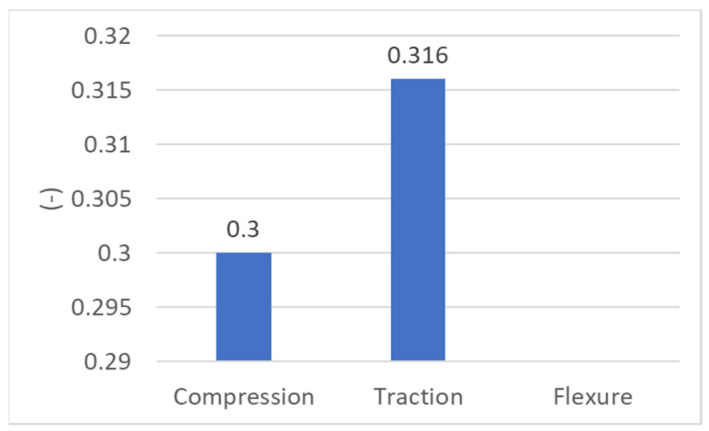
The values of the transverse shrinkage coefficient of the studied PLA.

Figure 46 shows the values of the longitudinal modulus of elasticity, determined as a result of the three tests performed.

**Figure 47:**
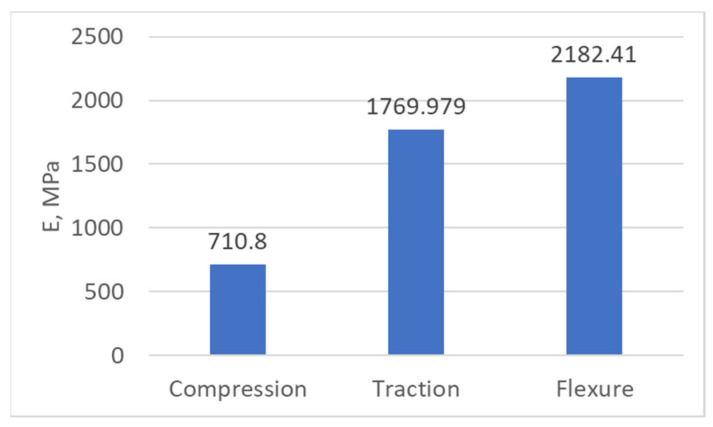
The values of the longitudinal modulus of elasticity of the PLA.

**Figure 48:**
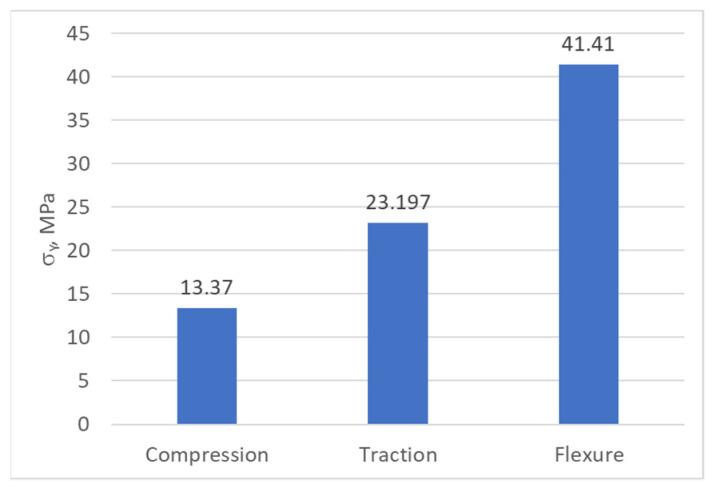
The yield strength values of the PLA.

**Figure 49:**
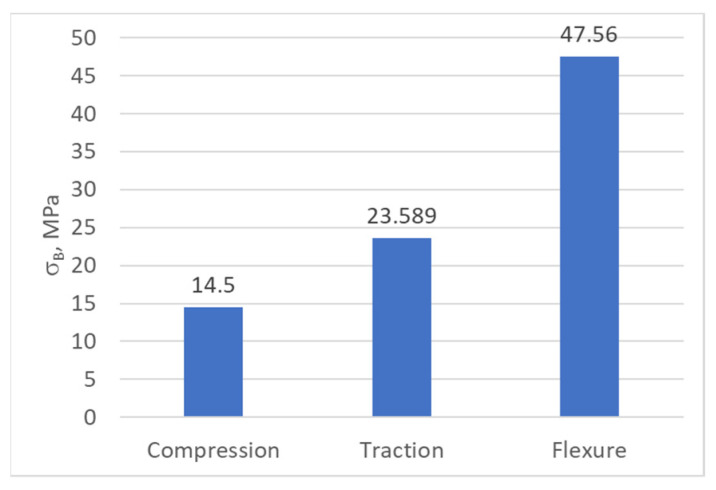
The breaking point values of the PLA.

The yield strength and fracture strength values, obtained via the mechanical tests performed, are presented in Figure 47, Figure 48 and Figure 49.

One conclusion of the mechanical results is that, due to the way the prosthesis is printed (the degree of filling), following the tests performed, the behavior of the material is different depending on the type of request. Through analyzing the obtained results, it can be concluded that the prosthesis withstands static and dynamic conditions without any problem.

Another conclusion would be that, instead of single-axis servomotors, we should use dual-axis servomotors, which would allow a cylindrical joint on both sides of the yoke. This will lead to an increased stiffness in the assembly. We will also further analyze the possibility of creating a coaxial passive joint with the servomotor axis on the opposite side of the yoke. These constructive variants will increase the rigidity of the system, reducing the level of vibrations.

For a better replication of the lower limb, we will try to re-smooth the fingers of the prosthesis (especially for the joint area near the toes), using the topology optimization method [29]. We will use attached springs, so that we can mimic their elasticity or stiffness, which is necessary in order to be able to simulate bipedal walking.

To determine the effort in the prosthesis that we will make (based on the model described), we will use a force sensor based on metal ionic composites (IP-MCs) [34], which are smart transducers made of materials that bend in response to low-voltage stimuli, and generate voltage in response to bending.

## Figures and Tables

**Table 1:** Physical and mechanical properties of PLA ^1^ [33].

Properties	Unit	PLA
	g/cm^3^	1.21–1.25
σ	MPA	21–60
E	GPA	0.35–3.5
ε	%	2.5–6
Σ *	Nm/g	16.8–48.0
E *	kNm/g	0.28–2.80
Tg	°C	45–60
Tm	°C	150–162

^1^—polymer density, σ—tensile strength, E—tensile modulus, ε—ultimate strain, Σ *—specific tensile strength, E *—specific tensile modulus, Tg—glass transition temperature, and Tm—melting temperature.

**Table 2:** The printing characteristics of the leg specimens and components are as follows.

Characteristics		Unit
Layer height		0.15 mm
First layer height		0.2 mm
Perimeters		2 lines
Solid layers	Top	8 layers
	Bottom	5 layers
Temperature	Nozzle	210 °C
	Bed	60 °C
Infill	Fill density	30%
	Fill pattern	Gyroid
Max volumetric speed		15 mm^3^/s

**Table 3:** The specimen dimensions required for compression.

Properties	Diameter, mm	Height, mm
PLA 1	19.93	19.98
PLA 2	19.87	19.97
PLA 3	19.92	20.01
PLA 4	19.89	19.98
PLA 5	19.85	19.99
PLA 6	19.86	20.00
PLA 7	19.86	19.98
PLA 8	19.87	20.00
PLA 9	19.91	19.98
PLA 10	19.89	19.99
Average, mm	19.885	19.998
Coefficient of variation, %	0.139	0.062

**Table 4:** The specimen dimensions required for traction.

Properties	Width, mm	Thickness, mm
PLA 1	10.02	4.94
PLA 2	10.02	4.96
PLA 3	9.99	4.90
PLA 4	9.99	4.92
PLA 5	9.99	4.91
PLA 6	10.02	4.93
PLA 7	10.02	4.92
PLA 8	10.02	4.91
PLA 9	10.01	4.95
PLA 10	10.02	4.91
Average, mm	10.010	4.925
Coefficient of variation, %	0.141	0.398

**Table 5:** The specimen dimensions required for bending.

Properties	Width, mm	Thickness, mm
PLA 1	19.90	4.96
PLA 2	19.94	4.91
PLA 3	19.86	4.99
PLA 4	19.90	4.96
PLA 5	19.86	4.97
PLA 6	19.96	4.97
PLA 7	19.88	4.95
PLA 8	20.01	4.91
PLA 9	19.93	4.98
PLA 10	19.89	4.94
Average, mm	19.913	4.954
Coefficient of variation, %	0.238	0.548

**Table 6:** The calf and sole mass characteristics.

Leg Component	The X-Coordinate of the Center of Mass Relative to the Local Frame (mm)	The Y-Coordinate of the Center of Mass Relative to the Local Frame (mm)	Mass (kg)
Calf	0.72	100.45	0.757
Sole	36.39	33.60	0.167

**Table 7:** The accelerometer positions relative to the sole-bound system.

Position	Coordinate x_2_ (mm)	Coordinate y_2_ (mm)
Position 1—on the axis of the joint	0	0
Position 2—in the front part of the sole	105	35.51

**Table 8:** Specimen dimensions required for bending.

Characteristic	Unit	Value
Distance between columns	mm	455
Distance between the lower and upper tank	mm	maxim 820
Force cell	kN	±25
Piston stroke	mm	±50 (100)
Sample holder system		hydraulic

**Table 9:** Specimen dimensions required for bending.

Characteristic	Unit	Value
Rooms	buc	2
Resolution	Mpx	5
Sensor size		2/3″
Frame rate	Hz	Up to 560
Communication		USB 3.0

**Table 10:** The mechanical characteristics of the tensile specimens.

Specimen	ν	Longitudinal Modulus of Elasticity, MPa	Drip Limit, MPa	Breaking Limit, MPa
PLA 1	0.304	1818.763	28.706	29.246
PLA 2	0.298	1677.115	22.027	22.425
PLA 3	0.322	1674.872	22.822	22.936
PLA 4	0.324	1766.250	22.910	23.044
PLA 5	0.303	1672.996	21.326	21.565
PLA 6	0.288	2317.956	22.678	24.460
PLA 7	0.370	1750.915	22.740	23.075
PLA 8	0.316	1687.123	22.895	22.988
PLA 9	0.316	1662.154	22.452	22.543
PLA 10	0.316	1671.644	23.414	23.607
Median	0.316	1769.979	23.197	23.589
Coefficient of variation, %	7.031	11.272	8.695	9.010

**Table 11:** The mechanical characteristics of the samples subjected to compression.

Specimen	ν	Longitudinal Modulus of Elasticity, MPa	Drip Limit, MPa	Breaking Limit, MPa
PLA 1	0.293	700.948	13.685	14.466
PLA 2	0.273	733.401	12.845	14.634
PLA 3	0.360	692.464	13.831	14.645
PLA 4	0.279	710.637	12.513	14.488
PLA 5	0.293	705.894	13.683	14.583
PLA 6	0.306	700.088	13.390	14.385
PLA 7	0.287	701.966	13.525	14.537
PLA 8	0.311	716.933	13.552	14.440
PLA 9	0.290	715.457	13.134	14.315
PLA 10	0.307	730.164	13.555	14.546
Median	0.300	710.795	13.371	14.504
Coefficient of variation, %	8.141	1.873	3.121	0.731

**Table 12:** The mechanical characteristics of the samples subjected to compression.

Specimen	Longitudinal Modulus of Elasticity, MPa	Drip Limit, MPa	Breaking Limit, MPa
PLA 1	2181.526	41.781	48.424
PLA 2	2212.134	43.496	46.498
PLA 3	2112.032	40.056	47.331
PLA 4	2161.289	41.159	48.424
PLA 5	2234.892	43.913	50.014
PLA 6	2182.306	39.152	46.252
PLA 7	2170.113	44.762	49.596
PLA 8	2204.104	43.626	48.519
PLA 9	2163.171	38.326	42.939
PLA 10	2229.581	37.838	47.601
Median	2182.415	41.411	47.560
Coefficient of variation, %	1.809	6.033	4.253

## Data Availability

Not applicable.
